# Metabolites Profiling and In Vitro Biological Characterization of Different Fractions of *Cliona* sp. Marine Sponge from the Red Sea Egypt

**DOI:** 10.3390/molecules28041643

**Published:** 2023-02-08

**Authors:** Wafaa H. B. Hassan, Zeinab I. El Sayed, Lamya H. Al-Wahaibi, Mahmoud M. Abdel-Aal, Wael M. Abdel-Mageed, Eman Abdelsalam, Sahar Abdelaziz

**Affiliations:** 1 Department of Pharmacognosy, Faculty of Pharmacy, Zagazig University, Zagazig 44519, Sharqia, Egypt; 2 Department of Pharmacognosy, Faculty of Pharmacy, Sinai University, Al-Arish 45511, North Sinai, Egypt; 3 Department of Chemistry, College of Science, Princess Nourah Bint Abdulrahman University, Riyadh 11671, Saudi Arabia; 4 Department of Pharmacognosy, College of Pharmacy, King Saud University, Riyadh 11451, Saudi Arabia; 5 Department of Pharmacognosy, Faculty of Pharmacy, Assiut University, Assiut 71526, Assiuṭ Governorate, Egypt

**Keywords:** UPLC-ESI-MS/MS, Red Sea, marine sponge, *Cliona* sp., secondary metabolites, antimicrobial, antioxidant, cytotoxicity

## Abstract

Red Sea marine sponges are an important source of biologically active natural products. Therefore, the present study aimed to investigate, for the first time, the components of *n*-hexane, dichloromethane, and ethyl acetate fractions of *Cliona* sp. marine sponge collected from the Red Sea, Egypt using UPLC-ESI-MS/MS (Ultra-performance liquid chromatography electrospray ionization tandem mass spectrometry) analysis. The analysis revealed the tentative identification of 23, 16, and 24 compounds from the *n*-hexane, dichloromethane, and ethyl acetate fractions of *Cliona* sp., respectively. In addition, the examination of these fractions resulted in the isolation and identification of three sterols and one amino acid. The identification of the isolated compounds was confirmed by 1D and 2D NMR (Nuclear Magnetic Resonance), and MS (Mass spectrometry), and IR (Infrared) spectroscopy. The in vitro cytotoxic, antioxidant, and antimicrobial activities of the total ethanolic extract and its sub-fractions were also evaluated. Interestingly, the ethyl acetate fraction showed potent cytotoxic activity against colon (HCT-116) and human larynx carcinoma (HEP-2) cell lines with IC_50_ (Half-maximal Inhibitory Concentration) 6.11 ± 0.2 and 12.6 ± 0.9 µg/mL, respectively. However, the dichloromethane fraction showed strong antioxidant activity, with IC_50_ 75.53 ± 3.41 µg/mL. Notably, the total ethanolic extract showed the strongest antibacterial activity against *Staphylococcus aureus* and *Escherichia coli*, with MIC (Minimum Inhibitory Concentration) 62.5 ± 0.82 and 125 ± 0.62 µg/mL, respectively, compared to other fractions. In conclusion, this is the first report on the secondary metabolites content and biological activities of *Cliona* sp. from the Red Sea, Egypt. It also highlights the need for further research on the most active fractions against various cancer cell lines and resistant bacterial and fungal strains. *Cliona* sp. extract and its fractions could be a potential source of novel and safe natural drugs with a wide range of medicinal and pharmaceutical applications.

## 1. Introduction

The Red Sea is a valuable and variant biosphere. Around 2000 km of coral reef runs along the Red Sea’s coastline, which is inhabited by various invertebrate species. The huge biodiversity and limited research make the Red Sea an unexplored goldmine for the innovation of novel biologically active marine drugs. Fifty-eight percent of the reported marine organisms are collected from Egypt due to the great biodiversity of the Egyptian environment. Notably, marine sponges are the most frequently collected organisms either from the Red Sea or around the world [1,2]. Sponges that belong to the animal kingdom, subkingdom parazoan, phylum porifera, are the oldest primitive multicellular organisms (dating back millions of years) with porous bodies for filtering their nutrients from water. They have variable shapes, sizes, and colors. About 10,000 known sponge species are present at different depths, either in fresh or marine waters, and serve as habitats for different organisms [3,4,5].

Sponges are sessile animals that have no physical defense and are easily attacked by various marine predators; therefore, they produce a wealth of defensive secondary metabolites with unique structures to forfend these predators and prevent the offshoots of tiny plants from adhering to them [3]. Recent research indicates that most of the bioactive metabolites from marine organisms have outstanding potency as antimicrobial, anti-inflammatory, anticancer, and immunostimulant drugs [6]. Natural compounds isolated from marine sponges may offer a proper means of developing unique therapeutic approaches for the management of variable diseases [7].

*Cliona* belongs to the family Clionaidae and comprises about 80 species spread worldwide on calcareous substrates, including coralline algae, coral, rocks, and shells. They contribute significantly to the erosive process that removes the calcium carbonate layer*. Cliona* sp secondary metabolites are expected to support *Cliona* sponges chemical defenses against fouling organisms, generalist fish predators, and those competing with corals for space [8]. *Cliona tenuis* contains various compounds, especially Clionapyrrolidine A, which can kill *Acropora palmata* (stony coral) tissues [9]. Although the genus has not received much chemical research, the few studies that have been done on it so far have shown that it contains a variety of natural product classes, including steroids, alkaloids, and peptides with variable biological activities [10]. Investigation of *Cliona viridis* from the Morocco Atlantic coast by GC-MS revealed various fatty acids such as stearic, palmitoleic, palmitic, oleic, and linoleic acids as well as variable sterols (campesterol, brassicasterol, β-sitosterol, cholesterol, avenasterol, and stigmasterol) [11]. *Cliona nigricans* contain clionastatins A and B, which are polyhalogenated steroids with significant cytotoxic activity [12].

*Cliona celata* collected from South Africa has potent anticancer activity due to the presence of aminosteroids and clionamines A–D [13]. There are several reports on the isolation of different peptide compounds from *Cliona* species such as tetraacetyl clionamide, clionamide, and celenamides A–D (linear peptide alkaloids) from the Canadian *Cliona celata* [14,15]. Storniamides A–D and celenamide E (tripeptide alkaloid) were isolated from Patagonian *Cliona* sp. and *Cliona chilensis*, respectively, and showed antibacterial activity against Gram-positive bacteria [16,17]. A detailed study of *Cliona patera* secondary metabolites resulted in the isolation of two macrolides and two alkaloids [18].

Based on the available literature, nothing was reported about the *Cliona* sp. collected from the Red Sea, Egypt, except by a study that described the isolation, metabolic profile, and biological properties of endozoic fungi derived from this sponge [19]. This motivated us to carry out the current work to investigate the chemical constituents of *Cliona* sp. using UPLC-ESI-MS/MS analysis along with the isolation and identification of the major components, in addition to the evaluation of their biological activities.

## 2. Results and Discussion

### 2.1. Characterization of the Isolated Compounds

**Figure 1:**
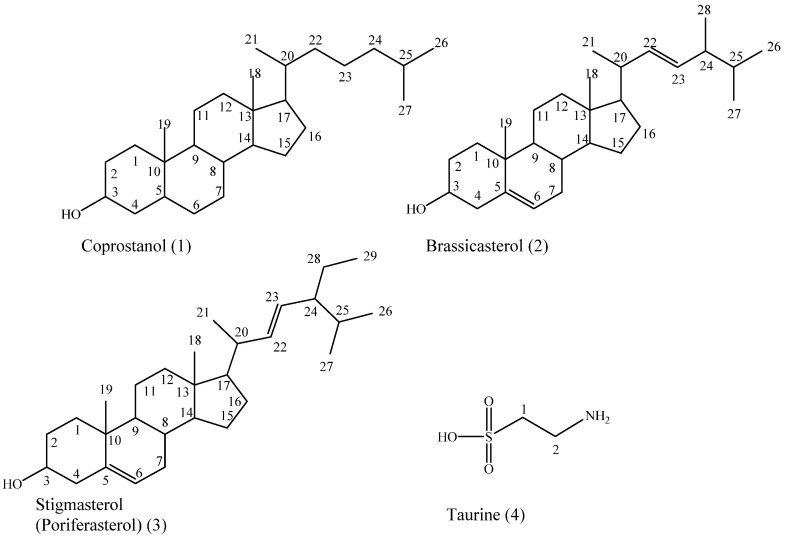
The chemical structures of compounds isolated from *Cliona* sp.

A secondary metabolites investigation of the Red Sea *Cliona* sp. revealed the isolation of four compounds. Various spectral analyses such as EI-MS, GC-MS, ^1^H-NMR, and ^13^C-NMR were used for the structure elucidation of the isolated compounds in addition to the comparison with the available works of literature. They were characterized as coprostanol (**1**), brassicasterol (**2**) [20,21,22], stigmasterol (**3**) [23], and taurine (**4**) [24,25] (Figure 1). To the best of our knowledge, this is the first report about the isolation of these compounds from *Cliona* sp.

Compounds **1** and **2** were obtained as a white amorphous mixture and were identified as coprostanol and brassicasterol based on a comparison of their spectroscopic analysis data, including EI-MS (Figure S1), GC-MS (Figures S2–S4), the ratio of coprostanol to brassicasterol (88.5% to 11.5%), ^1^H-NMR, and ^13^C-NMR (Table S1), with the reported literature data [20,21,22].

Compound **3** was isolated as a crystalline white powder with a melting point of 162 °C and was freely soluble in dichloromethane. It was identified as stigmasterol based on a comparison of its EI-MS (Figure S5), ^1^H-NMR, and ^13^C-NMR (Table S2) with the reported data [23].

Compound **4** is colorless needle crystals with a melting point of 300 °C and is soluble in hot methanol. It was identified as taurine based on a comparison of its EI-MS (Figure S6), ^1^H-NMR, and ^13^C-NMR with the reported literature [24,25].

### 2.2. Tentative Identification of Constituents of Cliona sp. Fractions by UPLC-ESI-MS/MS (Ultra-Performance Liquid Chromatography Electrospray Ionization Tandem Mass Spectrometry)

In the current study, UPLC-ESI-MS/MS in positive ion mode was used to analyze *Cliona* sp. From the Red Sea, Egypt *n*-hexane (CH), dichloromethane (CD), and ethyl acetate (CE) fractions. The compounds’ identification was based on their MS^2^ data given by the precursor ion mass, their fragments, and their neutral mass loss, as well as comparison with the available literature. Additionally, investigation of unidentified peaks from the total extract and fractions is currently underway.

#### 2.2.1. Characterization of the Components of *Cliona* sp. *n*-Hexane Fraction

**Figure 2:**
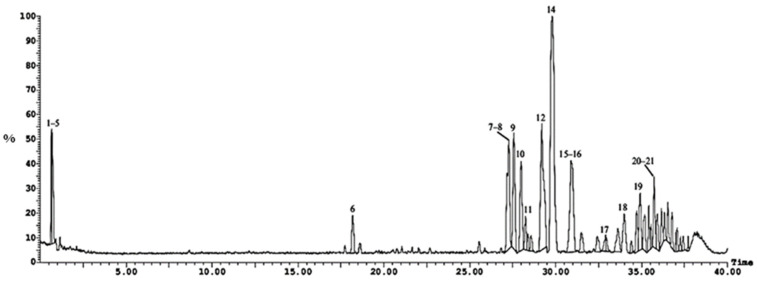
UPLC-ESI-MS chromatogram of *Cliona* sp. *n-*hexane, fraction in positive (+) ionization mode, numbers 1–21 refer to the tentatively identified compounds in *n*-hexane, fraction.

**Figure 3:**
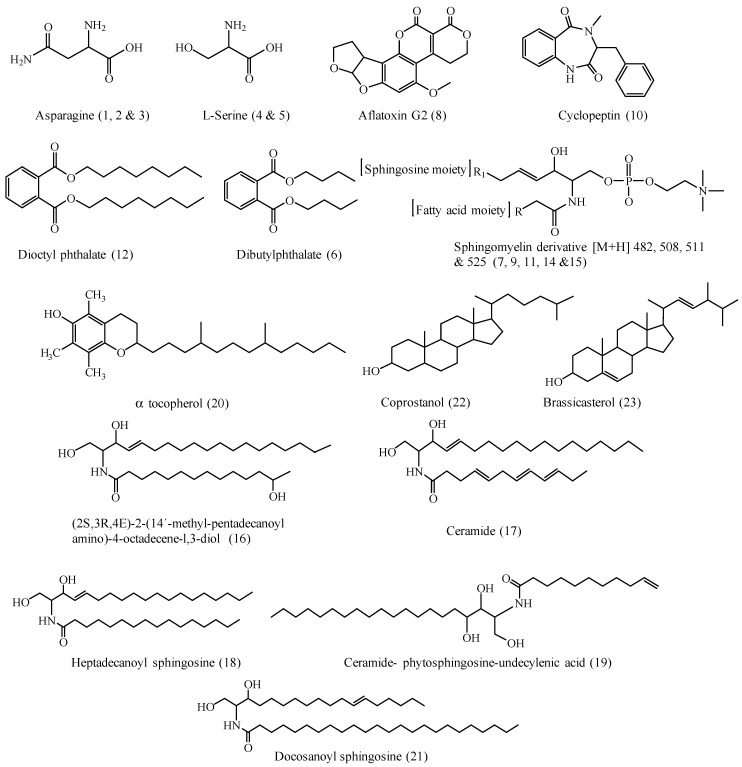
Chemical structures of the tentatively identified compounds from *Cliona* sp. *n*-hexane fraction.

Twenty-three compounds were tentatively identified by UPLC-ESI-MS/MS (positive ionization mode) from the *n*-hexane fraction of *Cliona* sp. Figure 2 and Figure 3 and Table 1 show the list of the identified compounds along with their retention time (R_t_), detected mass in positive ionization mode [M + H]^+^, and MS/MS fragment ions.

Compounds **1**, **2**, and **3** (R_t_. 0.48, 0.66, and 0.67 min, respectively) generated a protonated molecular ion peak at *m*/*z* 133 [M + H]^+^. They also gave a diagnostic MS^2^ fragment ion at *m*/*z* 88 [M + H-COOH]^+^ corresponding to the loss of the carboxylic group, in addition to the fragment ion at *m*/*z* 73 for C_2_H_4_NO_2_, showing the subsequent loss of COOH, NH_2_ moieties. According to the literature, compounds **1**, **2**, and **3** are tentatively identified as asparagine and its two isomers [26].

**Figure 4:**
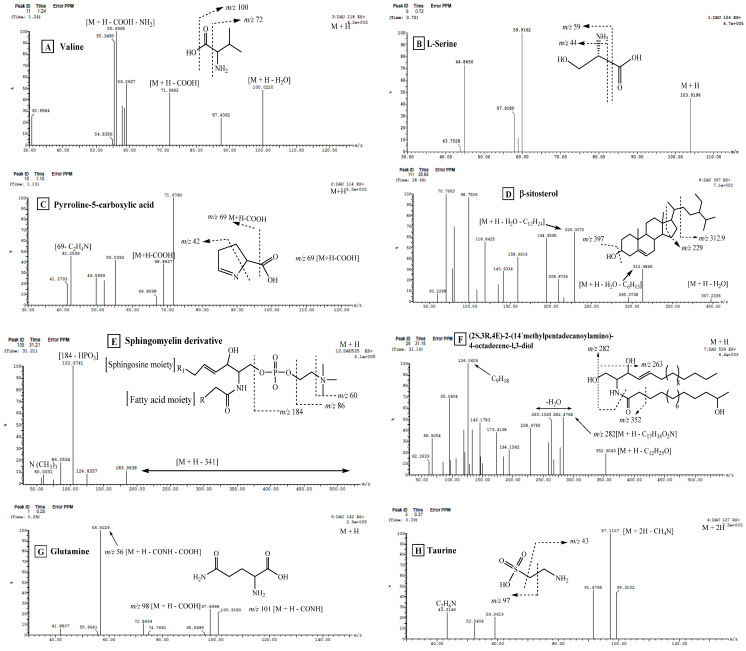

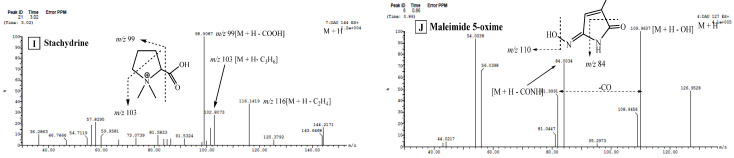
UPLC-ESI-MS/MS chromatograms of some identified compounds from *Cliona* sp. in positive (+) ionization mode: (**A**) Valine; (**B**) l-Serine; (**C**) Pyrroline-5-carboxylic acid; (**D**) β-sitosterol; (**E**) sphingomyelin derivative; (**F**) (2*S*, 3*R*, 4*E*)-2-(14′-methyl-pentadecanoylamino)-4-octadecene-l,3-diol; (**G**) Glutamine; (**H**) Taurine; (**I**) Stachydrine; (**J**) Maleimide 5-oxime.

Compounds **4** and **5** (R_t_. 0.72 and 0.83 min, respectively) gave similar ESI-MS^1^ at *m*/*z* 104 [M + H]^+^ and ESI-MS^2^ fragment ion at *m*/*z* 59 [M + H-COOH]^+^, showing the loss of a carboxyl group; this is considered to be the base peak (Figure 4B). Furthermore, fragment ion at *m*/*z* 43 [59-NH_2_]^+^ corresponds to the loss of the amino group. With these data, with the aid of the literature, compounds 4 and 5 were tentatively identified as L-serine with its isomer [26].

Compound **6** (R_t_. 18.18 min) showed ESI-MS^1^ at *m*/*z* 301 [M + Na]^+^ and *m*/*z* 279 [M + H]^+^, and fragment ion at *m*/*z* 149, which showed a loss of 130 amu corresponding to C_4_H_9_ and C_4_H_9_O moieties. Accordingly, this compound was tentatively identified as dibutyl phthalate [27,28].

Compound **7** (R_t_. 27.38 min) exhibited ESI-MS^1^ at *m*/*z* 482 [M^+^ + H]^+^. The MS^2^ showed a daughter ion at *m*/*z* 184, corresponding to protonated phosphocholine moiety. The base peak fragment at *m*/*z* 104 [184-HPO_3_]^+^ showed a loss of phosphate moiety from the phosphocholine base. The fragment ion at *m*/*z* 124 was assigned for the loss of N(CH_3_)_3_ from phosphocholine moiety, which was confirmed by the presence of the fragment ion at *m*/*z* 60. Unfortunately, ions reflecting the fatty acid constituent and the identities of the sphingosine base were not observed as it was cleaved together, leaving phosphocholine moiety; therefore, compound **7** was tentatively identified as a sphingomyelin derivative [29].

Compound **8** (R_t_. 27.74 min) was tentatively identified as aflatoxin G. It generated ESI-MS^1^ at *m*/*z* 353 [M + Na]^+^ and MS^2^ fragment ion at *m*/*z* 295 [M + H-OCH_3_-CO]^+^, corresponding to the loss of a methoxy and carbonyl groups. Additionally, a daughter ion at *m*/*z* 275 showed the loss of two carbonyl groups beside sodium. Furthermore, the fragment ion at *m*/*z* 243 showed the loss of two oxygen atoms from 275 amu, while the fragment ion at *m*/*z* 228 showed the subsequent loss of the CH_3_ group. Through comparison with the available literature, compound **8** was tentatively identified as aflatoxin G2 [30,31,32].

Compound **10** (R_t_. 28.08 min) showed an ESI-MS protonated molecular ion peak at *m*/*z* 281 [M + H]^+^. The cleavage of the seven-membered ring gave two important fragment ions at *m*/*z* 120 and 161. Both fragments subsequently gave a fragment ion at *m*/*z* 104 amu after the loss of NH from the fragment ion at *m*/*z* 120 amu and the loss of CO and NCH_3_ from the other fragment at *m*/*z* 161. Additionally, another fragment ion was observed at *m*/*z* 223 [M + H-CONCH_3_]^+^ which, by the loss of benzoyl moiety, gave a characteristic fragment ion at *m*/*z* 133 amu. The mass data of this compound are in good agreement with the reported literature on cyclopeptin [33].

Compounds **9**, **11**, and **13** (R_t_. 27.79, 29.07, and 29.52 min, respectively) exhibited ESI-MS at *m*/*z* 508 and 511 [M + H]^+^, respectively. MS^2^ showed the same fragmentation pattern as compound **7**; therefore, the two compounds were tentatively identified as sphingomyelin derivatives [29].

Compound **12** (R_t_. 29.20 min) showed ESI-MS^1^ at *m*/*z* 413 [M + Na]^+^ and *m*/*z* 391 [M + H]^+^. Fragment ion was observed at *m*/*z* 279 due to neutral loss of C_8_H_16_. Another important fragment ion at *m*/*z* 149 for phthalate moiety was produced after the loss of C_8_H_17_ and C_8_H_17_O moieties. Accordingly, this compound was tentatively identified as dioctyl phthalate [27].

Compounds **14** and **15** (R_t_. 30.65 and 31.18 min, respectively) exhibited ESI-MS^1^ at *m*/*z* 525 [M + H]^+^. The MS^2^ fragmentation pattern was similar to that of compound **7**; therefore, compounds **14** and **15** were tentatively identified as sphingomyelin derivatives (Figure 4E) [29].

Compound **16** (R_t_. 31.19 min) was tentatively identified as (2*S*,3*R*, 4*E*)-2-(14′-methyl-pentadecanoylamino)-4-octadecene-l,3-diol (Figure 4F). It generated ESI-MS^1^ at *m*/*z* 539 [M + H]^+^. The MS^2^ showed a fragment ion at *m*/*z* 352 [M + H-C_12_H_25_O]^+^, which resulted from the loss of fatty acid moiety, and *m*/*z* 282 [M + H-C_15_H_30_O_2_N]^+^ corresponding to a sphingosine part after the neutral loss of one water molecule. In addition, the fragment ion at *m*/*z* 264 showed a subsequent loss of water molecules from sphingosine moiety. The base peak fragment at *m*/*z* 126, corresponding to C_9_H_18_ moiety, was also detected. The data of this compound are in good agreement with those of (2*S*,3*R*,4*E*)-2-(14′-methyl-pentadecanoylamino)-4-octadecene-l,3-diol [34].

Compound **17** (R_t_. 33.21 min) exhibited a protonated molecular ion peak at *m*/*z* 476 [M + H]^+^ and MS^2^ fragment ions at *m*/*z* 329 [M + H-C_11_H_17_]^+^ and 299 [329-CH_2_OH]^+^, and 281 [299-H_2_O]^+^. Furthermore, a fragment ion at *m*/*z* 126 corresponding to C_9_H_19_ was also observed. According to these data, and with the aid of the available literature, compound **17** was tentatively identified as ceramide with a sphingosine base [C18:3] and a fatty acid base [C12:1] [29,34].

Compound **18** (R_t_. 34.09 min) was tentatively identified as *N*-heptadecanoyl sphingosine. It generated ESI-MS^1^ at *m*/*z* 551 [M + H]^+^ and an MS^2^ fragment ion at *m*/*z* 299 [M + H-C_17_H_35_O]^+^, corresponding to the sphingosine moiety after the loss of the fatty acid part (heptadecanoic acid). The MS data are in good agreement with the literature for *N*-heptadecanoyl sphingosine [35].

Compound **19** (R_t_. 35.37 min) generated a molecular ion peak [M^+^] at *m*/*z* 483. Its MS^2^ gave a diagnostic fragment ion at *m*/*z* 317 [M + H-C_11_H_18_O]^+^, corresponding to the phytosphingosine moiety after the loss of fatty acid moiety (undecylenic acid). Furthermore, the fragment ion at *m*/*z* 255 [317-NH-CH_2_OH-OH]^+^ showed a loss of OH, CH_2_OH, and NH groups from the phytosphingosine part. Accordingly, this compound was tentatively identified as ceramide with phytosphingosine and undecylenic acid moiety [35].

Compound **20** (R_t_. 36.77 min) was tentatively identified as *α*-tocopherol. It generated ESI-MS^1^ at *m*/*z* 431 [M + H]^+^. The MS^2^ fragment ion at *m*/*z* 377 amu showed the loss of C_4_H_6_ isobutyl moiety from the side chain. The base peak fragment at *m*/*z* 190.9 [M + H-C_17_H_37_]^+^, corresponding to the loss of the side chain beside another methyl group, was detected. Furthermore, fragment ions at *m*/*z* 149, 136, 120 [136-CH_3_]^+^, and 97 were also observed. These data, in addition to comparison with literature, led to the tentative identification of compound **20** as *α*-tocopherol [36,37].

Compound **21** (R_t_. 36.78 min) exhibited a molecular ion peak at *m*/*z* 648 [M + H]^+^. It also gave a diagnostic MS^2^ fragment ion at *m*/*z* 365 [M + H-C_24_H_46_ON]^+^, corresponding to the loss of docosanoic acid (22:0) and C_2_H_4_N. Furthermore, fragment ions at *m*/*z* 125 (C_9_H_17_), 97 (C_7_H_13_), and 71 (C_5_H_11_) were also observed. The fragment ion at *m*/*z* 97 revealed the presence of a double bond at C6 in sphingosine moiety. Therefore, compound **21** was tentatively identified as docosanoyl sphingosine [34,38].

Compound **22** (R_t_. 37.00 min) exhibited a molecular ion peak at *m*/*z* 371 [M + H-H_2_O]^+^, corresponding to C_27_H_48_O after the loss of one water molecule. In comparison with the literature [39], compound **22** was identified as coprostanol.

Compound **23** (R_t_. 37.43 min) exhibited a molecular ion peak at *m*/*z* 399 [M + H]^+^ and *m*/*z* 381 [M + H-H_2_O]^+^, corresponding to C_28_H_46_O after the loss of one water molecule. In comparison with the literature [21], compound **23** was identified as brassicasterol.

#### 2.2.2. Characterization of the Components of *Cliona* sp. Dichloromethane Fraction

**Figure 5:**
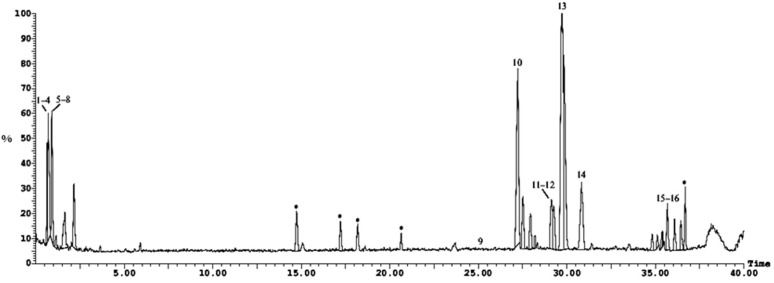
UPLS-ESI-MS chromatogram of dichloromethane fraction in positive (+) ionization mode. * Unidentified compounds, numbers 1–16 refer to the tentatively identified compounds in dichloromethane fraction.

**Figure 6:**
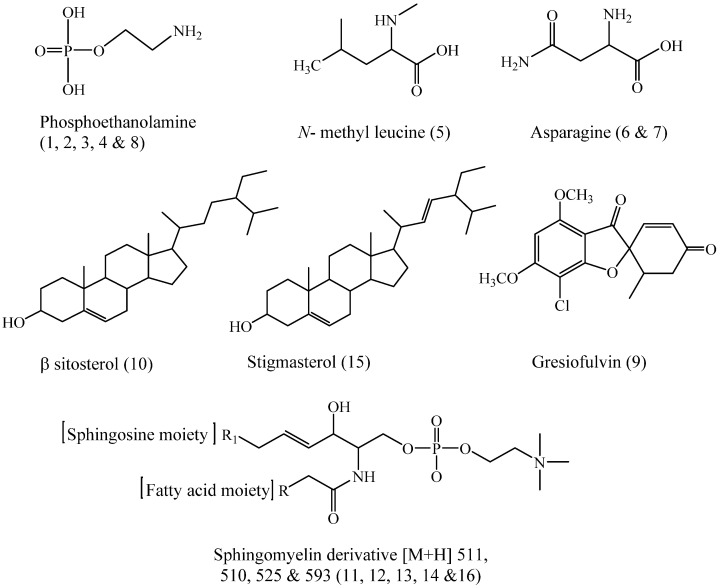
Chemical structures of the tentatively identified compounds from dichloromethane fraction of *Cliona* sp.

The HPLC-ESI-MS/MS analysis of the methylene chloride fraction of the Red Sea sponge *Cliona* sp. led to the tentative identification of 15 compounds, as shown in Figure 5, Figure 6 and Table 2.

Compounds **1**–**4** and **8** (R_t_. 0. 14, 0.23, 0.31, 0.41, and 0.92 min, respectively) were tentatively identified as phosphoethanolamine and its isomers from ESI-MS at *m*/*z* 142 [M + H]^+^. The MS^2^ fragmentation gave abundant daughter ions at *m*/*z* 82 for [M + H-C_2_H_6_NO]^+^, corresponding to the loss of ethanolamine leaving H_2_PO_3_ moiety in addition to other fragments at *m*/*z* 124 [M + H-H_2_O] ^+^ and 108 [M + H-NH_2_-H_2_O]^+^, which were also observed. These data confirmed that compounds **1**–**4** and **8** were phosphoethanolamines with four stereoisomers [40].

Compound **5** (R_t_. 0. 74 min) showed a molecular ion peak at *m*/*z* 146 [M + H]^+^ and a MS^2^ high abundant fragment ion at *m*/*z* 103 [M + H-C_3_H_7_]^+^ for the loss of isopropyl in addition to 86 [M + H-COOH-CH_3_]^+^, corresponding to the loss of carboxyl and methyl groups. Another fragment at *m*/*z* 59 [M + H-COO-C_3_H_7_]^+^ was also detected. Therefore, compound **5** was tentatively identified as *N*-methyl leucine [26].

Compounds **6** and **7** (Rt. 0.55 and 0.67 min, respectively) generated a protonated molecular ion peak at *m*/*z* 133 [M + H]^+^. They also gave diagnostic MS^2^ fragment ions at *m*/*z* 88 [M + H-COOH]^+^, which corresponded to the loss of the carboxylic group. In addition, the fragment ion at *m*/*z* 73 for C_2_H_4_NO_2_ showed the subsequent loss of C_2_H_4_NO moiety. According to the literature [41], compounds **6** and **7** were tentatively identified as asparagine and its isomer [26].

Compound **9** (R_t_. 25.16 min.) exhibited a protonated molecular ion peak at *m*/*z* 353 [M + H]^+^. MS^2^ ions were observed at *m*/*z* 321 and 257, corresponding to the loss of the methoxy group and chloride atom, respectively. A fragment at *m*/*z* 215 was observed, corresponding to C_8_H_7_O_4_Cl, while the fragments at *m*/*z* 165 are due to C_7_H_4_O_4_. Therefore, compound **9** was tentatively identified as griseofulvin [42].

Compound **10** (R_t_. 28.66 min) exhibited a molecular ion peak at *m*/*z* 397 [M + H-H_2_O]^+^, with daughter ions at *m*/*z* 312.9 [M + H-H_2_O-C_6_H_13_]^+^. In addition, ion fragments at *m*/*z* 229 [M + H-H_2_O-C_12_H_24_]^+^ corresponded to the loss of the side chain beside the C_2_H_3_ moiety (Figure 4D). With these data, and with the aid of the available literature, compound **10** was tentatively identified as β-sitosterol [43].

Compounds **11**, **12**, **14**, and **16** (R_t_. 29.07, 29.52, 31.21, and 35.67 min, respectively), exhibited ESI-MS^1^ at *m*/*z* 511, 510, 525, and 593 respectively, [M]^+^ and [M + H]^+^. The MS^2^ of all of these compounds showed daughter ions at *m*/*z* 184, corresponding to protonated phosphocholine moiety. The base peak fragment at *m*/*z* 104 [184-HPO_3_]^+^ showed a loss of phosphate moiety from the phosphocholine base. The fragment ion at *m*/*z* 124 was assigned for the loss of the N (CH_3_)_3_ moiety, which was confirmed by the presence of a fragment ion at *m*/*z* 60. Unfortunately, fragment ions reflecting the fatty acid constituent and the identities of the sphingosine base were not observed, as they were cleaved together, leaving phosphocholine moiety; therefore, the four compounds were tentatively identified as sphingomyelin derivatives [29].

Compound **13** (R_t_. 30.79) exhibited ESI-MS^1^ at *m*/*z* 547 [M + Na]^+^. The MS^2^ fragment at *m*/*z* 487 [M + H-N(CH_3_)_3_]^+^ showed a loss of N(CH_3_)_3_. Furthermore, the fragment ion at *m*/*z* 264 corresponded to the loss of fatty acid moiety (d 5:1) and two water molecules from the sphingosine base (C18:1). Moreover, fragment ions were observed at *m*/*z* 147 and 86 [CH_2_ = CH-N(CH_3_)_3_]^+^. These data confirmed that compound **13** is a sphingomyelin derivative with a sphingosine base (d18:1) and fatty acid (C5:1) [29].

Compound **15** (R_t_. 35.35) exhibited ESI-MS^1^ at *m*/*z* 395 [M + H-H_2_O]^+^, corresponding to C_29_H_48_O after the loss of the water molecule. From the previously mentioned data and through comparison with published data [23], compound **15** was identified as stigmasterol.

**Table 2:** Metabolites tentatively identified in dichloromethane fraction using UPLC-ESI-MS/MS analysis in positive ionization mode.

No.	Compound Name	R_t_ (min.)	Parent Ion (*m*/*z*)	MS^2^ Fragments (*m*/*z*)	Area % Total	Reference
1	Phosphorylethanolamine isomer	0.14	142 [M + H]^+^	124, 110, 85, 82, 78, 69, 66, 55, 52	2.84	[29]
2	Phosphorylethanolamine isomer	0.23	142 [M + H]^+^	124, 110, 85, 82, 78, 69, 66, 55, 52	1.20	[29]
3	Phosphorylethanolamine isomer	0.31	142 [M + H]^+^	124, 110, 85, 82, 78, 69, 66, 55, 52	0.74	[29]
4	Phosphorylethanolamine isomer	0.41	142 [M + H]^+^	124, 110, 85, 82, 78, 69, 66, 55, 52	1.30	[29]
5	*N*-methyl leucine	0.48	146 [M + H]^+^	131, 87	3.38	[26]
6	Asparagine	0.55	133 [M + H]^+^	88, 73	2.84	[26]
7	Asparagine isomer	0.67	133 [M + H]^+^	88, 73	5.73	[26]
8	Phosphorylethanol amine	0.92	142 [M + H]^+^	124, 110, 85, 82, 78, 69, 66, 55, 52	5.06	[26]
9	Gresiofulvin	25.16	353 [M + H]^+^	21, 257, 215, 165	12.83	[44]
10	β*-*Sitosterol	28.66	397 [M + H-18]^+^	312	11.68	[43]
11	Sphingomyelin derivative	29.07	511 [M + H]^+^	184, 124, 104, 60	21.48	[29]
12	Sphingomyelin derivative	29.52	510 [M + H]^+^	184, 124, 104, 60	3.23	[29]
13	Sphingomyelin derivative	30.79	547 [M + H]^+^	487, 264, 147, 86	2.69	[29]
14	Sphingomyelin derivative	31.21	525 [M + H]^+^	184, 124, 104, 60	6.05	[29]
15	Stigmasterol *	35.35	395 [M + H-H_2_O]		0.45	[23]
16	Sphingomyelin derivative	35.67	593 [M + H]^+^	184, 124, 104, 60	2.31	[45]

* Compounds isolated from dichloromethane fraction.

#### 2.2.3. Characterization of the Components of *Cliona* sp. Ethyl Acetate Fraction

**Figure 7:**
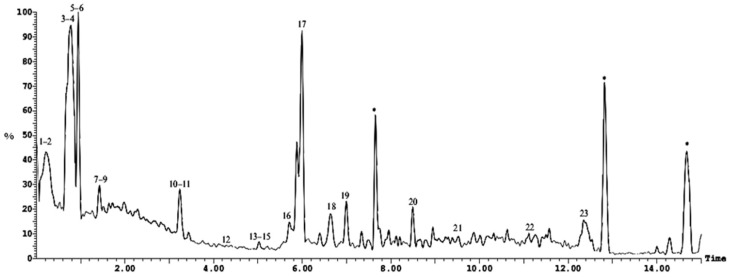
UPLS-ESI-MS chromatogram of ethyl acetate, fraction in positive (+) ionization mode * Unidentified compounds, numbers 1–24 refer to the tentatively identified compounds in ethyl acetate fraction.

**Figure 8:**
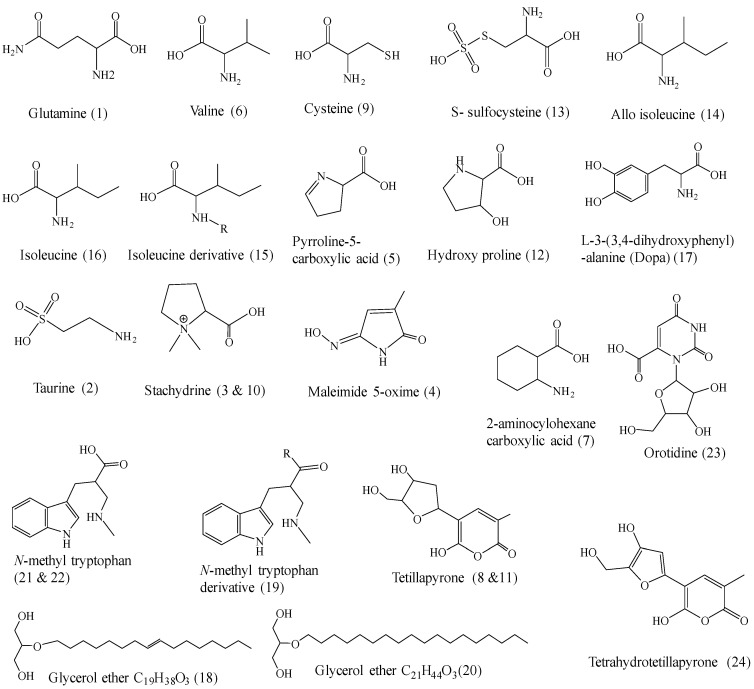
Chemical structures of the tentatively identified compounds from ethyl acetate fraction of *Cliona* sp.

The HPLC-ESI-MS/MS analysis of *Cliona* sp. ethyl acetate led to the tentative identification of twenty-four compounds, as shown in Figure 7, Figure 8 and Table 3.

Compound **1** (R_t_. 0.25 min) was tentatively assigned as glutamine (Figure 4G). It showed a protonated molecular ion peak [M + H]^+^ at *m*/*z* 142 and daughter ions at *m*/*z* 101 [ M + H-CONH]^+^. The neutral loss of the carboxyl group was confirmed by the presence of a fragment at *m*/*z* 56 [ M + H-CONH-COOH]^+^. From this fragmentation pattern, and through comparison with the literature, compound **1** was concluded to be glutamine [26].

Compound **2** (R_t_. 0.37 min) generated a protonated molecular ion at *m*/*z* 127 [M + 2H]^+^. The MS^2^ spectrum showed a base peak at *m*/*z* 97 [M + H-NH_2_CH_2_]^+^. In addition, the fragment ion at *m*/*z* 43 [M + 2H-SO_3_H]^+^, corresponding to the loss of the sulphate group was detected. These data were consistent with the data of the amino acid taurine (Figure 4H) (2-aminoethanesulfonic acid) [46].

Compounds **3** and **10** (R_t_. 0.76 and 3.02 min, respectively) generated a protonated molecular ion peak at *m*/*z* 144 [M + H]^+^. It gave diagnostic MS^2^ fragment ions at *m*/*z* 103 [M + H-C_3_H_6_]^+^, corresponding to ring cleavage. The base peak at *m*/*z* 99 [M + H-COOH]^+^, showing the loss of the carboxyl group, was produced (Figure 4I). According to the literature, compounds **3** and **10** were tentatively identified as stachydrine and its isomer [47].

Compound **4** (R_t_ 0.86 min) exhibited a protonated ESI-MS at *m*/*z* 127 [M + H]^+^. MS^2^ daughter ions at *m*/*z* 110 [M + H-H_2_O]^+^ showed a loss of a water molecule. In addition, the fragment ions at *m*/*z* 84 [M + H-CONH]^+^, 82 [109-CO]^+^, and 44 were detected. By comparison with the reported structural data, this compound was tentatively assigned as maleimide 5-oxime (Figure 4J), which was previously isolated from *Cliona Patera* [18].

Compound **5** (R_t_. 1.10 min) generated a molecular ion peak at *m*/*z* 114 [M + H]^+^, and the MS^2^ spectrum (Figure 4C) showed daughter ions at *m*/*z* 69 [M + H-COOH]^+^, corresponding to the loss of carboxylic acid. In addition, fragment ions at *m*/*z* 42 [69-CHN]^+^ showed a loss of CHN, while the base peak fragment at *m*/*z* 71 showed a loss of C_2_H_4_N. These data confirmed that compound **5** is pyroline-5 carboxylic acid [26].

Compound **6** (R_t_. 1.24 min) exhibited ESI-MS at *m*/*z* 118 [M + H]^+^ (Figure 4A). The MS^2^ spectrum showed daughter ions at *m*/*z* 100 [M + H-H_2_O]^+^, corresponding to the loss of the H_2_O molecule. Additionally, a fragment ion at *m*/*z* 72 [M + H-COOH]^+^ showed the loss of the COOH group of the amino acid, followed by loss of the amino group to give a fragment ion at *m*/*z* 56. From the previous data and by comparison with the available literature, compound **6** was tentatively identified as valine [48].

Compound **7** (R_t_. 1.27 min) was tentatively identified as 2-aminocyclohexane carboxylic acid from the ESI-MS and MS^2^ spectral data. It also showed a protonated molecular ion peak at *m*/*z* 144 [M + H]^+^ and daughter ions at *m*/*z* 128 [M + H-NH_2_]^+^, corresponding to the loss of the amino group, in addition to fragment ions at *m*/*z* 98 [M + H-COOH]^+^ due to the loss of the carboxylic group. These data are in good agreement with the available literature on 2-amino cyclohexane carboxylic acid [49].

Compounds **8** and **11** (R_t_. 1.39 and 3.24 min, respectively) were tentatively assigned as tetillapyrone with its stereoisomer. They showed ESI-MS molecular ion peaks at *m*/*z* 243, 485 [M + H]^+^, and [2M + H]^+^. They produced diagnostic fragment ions at *m*/*z* 197 [M + H-COOH]^+^. A base peak at *m*/*z* 84 [C_4_H_4_O_2_]^+^ of furan ring A was also observed. These data are in good agreement with the literature on tetillapyrone. It is worth noting that this compound was previously isolated from *Cliona Patera* [18].

Compound **9** (R_t_. 1.43 min) exhibited ESI-MS at *m*/*z* 122 [M + H]^+^, in addition to a fragment ion at *m*/*z* 104 [M + H-CO-H_2_O]^+^. According to these data, and with the help of the available literature, compound **9** was identified as cysteine [50].

Compound **12** (R_t_. 4.28 min) exhibited an ESI-MS at *m*/*z* 132 [M + H]^+^. The MS^2^ spectrum showed abundant daughter ions at *m*/*z* 86 [M + H-COOH]^+^, corresponding to the loss of carboxylic acid moiety. In addition, a fragment ion at *m*/*z* 56 showed the subsequent loss of the CH_4_N fragment; therefore, compound **12** was tentatively identified as hydroxy proline [51].

Compound **13** (R_t_. 5.02) exhibited positive ESI-MS at *m*/*z* 202 [M + H]^+^, in addition to fragment ions which were also detected at *m*/*z* 156 [M + H-CO-H_2_O]^+^. From these data, and with the help of the available literature, compound **13** was tentatively identified as *S*-sulfocysteine [26,51].

Compounds **14** and **16** (R_t_ 5.04 and 5.35 min, respectively) were tentatively identified as allo-isoleucine and isoleucine, as they showed molecular ion peaks in the ESI-MS spectrum at *m*/*z* 132 [M + H]^+^. MS^2^ exhibited daughter ions at *m*/*z* 86 [M + H-COOH]^+^, corresponding to the loss of the carboxyl group. Furthermore, the fragment ion at *m*/*z* 69 [M + H-COOH-NH_2_]^+^ showed a further loss of the amino group [26].

Compound **15** (R_t_ 5.05 min) was tentatively identified as an isoleucine derivative from ESI-MS and MS^2^ spectral data. The molecular ion peak was formed at *m*/*z* 216 and the MS^2^ spectrum showed fragment ions at *m*/*z* 132 [M + H-R]^+^, corresponding to the loss of R-moiety and give amu of isoleucine. The prominent base peak produced at *m*/*z* 86 [M + H-R-COOH]^+^ showed a loss of the carboxyl group, confirming the presence of isoleucine. According to these data, compound **15** was tentatively identified as an isoleucine derivative. [26].

Compound **17** (R_t_ 6.40) generated its ESI-MS molecular ion peak at *m*/*z* 198 [M + H]^+^. The MS^1^ fragment ion produced at *m*/*z* 144 showed the loss of three molecules of water in addition to fragment ions at *m*/*z* 111 due to the loss of the side chain C_3_H_6_O_2_N. From these data, and through comparison with the available literature, compound **17** was tentatively identified as L-3-(3,4-dihydroxyphenyl)-alanine (Dopa) [26].

Compound **18** (R_t_. 6.40 min) at negative mode exhibited a deprotonated molecular ion peak at *m*/*z* 313 [M−H]^−^. The fragment ion at *m*/*z* 227 showed a loss of C_6_H_13_ moiety. In addition, the fragment ion at *m*/*z* 173 showed a subsequent loss of C_4_H_6_ (54 amu.), indicating the presence of a double bond in this region. This suggestion was confirmed by the fragment ion at *m*/*z* 117 showing a subsequent loss of C_4_H_8_ (56 amu). Using these fragmentation patterns with the aid of the available literature, compound **18** was tentatively identified as glycerol ether with the molecular formula C_19_H_38_O_3_. Similar compounds were previously identified from *Cliona patera* [18]; the only difference was the presence of an extra double bond in our compound.

Compound **19** (R_t_. 7.19 min) exhibited ESI-MS in negative mode at *m*/*z* 309 [M]^−^, in addition to a prominent fragment ion at *m*/*z* 219 [M + H-R]^−^, corresponding to the molecular weight of *N*-methyl tryptophan. From these data and with the help of the available literature, compound **19** was tentatively identified as an *N-*methyl tryptophan derivative [52].

Compound **20** (R_t_. 7.33 min) showed an ESI-MS spectrum [M + H]^+^ at *m*/*z* 345 and daughter ions at *m*/*z* 316 [M + H-C_2_H_5_]^+^, corresponding to the loss of the ethyl group, and, at *m*/*z* 252 [M + H-C_4_H_9_-2H_2_O]^+^, due to loss of C_4_H_9_ and two molecules of water. In addition, the fragment ion at *m*/*z* 201 [M + H- C_9_H_18_-H_2_O]^+^ showed the loss of C_9_H_18_ moiety and one molecule of water, while the fragment at *m*/*z* 158 showed the loss of the C_12_H_24_ moiety and one molecule of water. Therefore, compound **20** was identified as glycerol ether with the molecular formula C_21_H_44_O_3_; this compound was previously isolated from *Cliona patera* [18].

Compounds **21** and **22** (R_t_. 8.92 and 9.44 min, respectively) were tentatively identified as *N*-methyl tryptophan and its stereoisomer. They generated ESI-MS^1^ at *m*/*z* 219 [M + H]^+^. The MS^2^ fragment showed fragment ions at *m*/*z* 188 [M + H-NHCH_3_]^+^, corresponding to the loss of the methyl amine group, and at 173 [M + H-COOH]^+^, due to the loss of the carboxyl group. In addition, the fragment ion at *m*/*z* 101 was due to the side chain C_4_H_7_O_2_N, while the fragment at *m*/*z* 118 was due to C_8_H_7_N. The mass data are in good agreement with the literature [52].

Compound **23** (R_t_. 11.11 min) generated a protonated molecular ion peak at *m*/*z* 289 [M + H]^+^. It gave diagnostic MS^2^ fragment ions at *m*/*z* 270 [M + H-H_2_O]^+^, 252 [M + H-2H_2_O]^+^, and 132 [M + H-C_5_H_9_O_4_]^+^, corresponding to the neutral loss of ribose sugar. The prominent base peak at *m*/*z* 89 showed a loss of C_2_H_2_N_2_O_2_ moiety. According to the literature, compound 23 was tentatively identified as orotidine [26].

Compound **24** (R_t_. 12.03) exhibited an ESI-MS^1^ protonated molecular ion peak at *m*/*z* 239 [M + H]^+^ in addition to the fragment ion at *m*/*z* 193 [M + H-COOH]^+^, which showed neutral cleavage of the carboxylic group. Furthermore, the fragment ion at *m*/*z* 113 [M + H-C_5_H_5_O_3_]^+^ corresponded to the loss of the lactone ring. Another fragment was observed at *m*/*z* 85 for C_4_H_4_O_2_ of the lactone ring with the OH group. This fragmentation helped in the tentative identification of compound **24** as tetradehydro tetillapyrone. It is worth noting that tetillapyrone was previously isolated from the sponge *Cliona patera* [18].

### 2.3. Biological Activities of Cliona sp. Total Extract (CT), n-Hexane (CH), Dichloromethane (CD) and Ethyl Acetate (CE) Fractions

#### 2.3.1. Antimicrobial activity and MIC *

**Figure 9:**
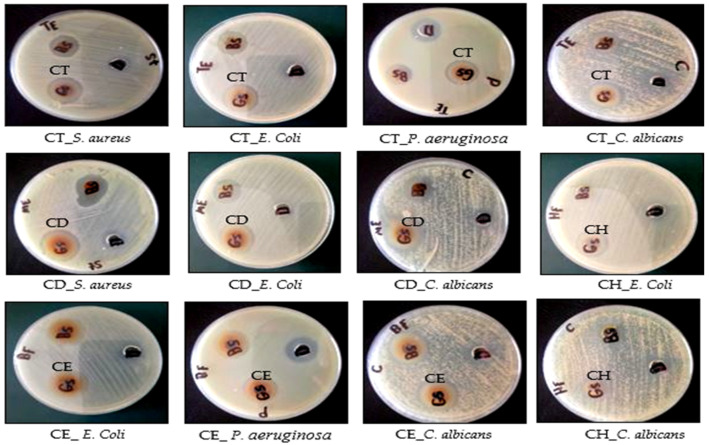
Antimicrobial activity of *Cliona* sp. Total extract (CT), *n*-Hexane (CH), Dichloromethane (CD), and Ethyl acetate (CE) fractions by agar dilution method with *S. aureus*, *E. coli*, *P. aeruginosa*, and *C. albicans*. Ciprofloxacin (antibacterial) and Fluconazole (antifungal) were used as positive controls while DMSO was the negative control.

In the present study, the antimicrobial activity of *Cliona* sp. fractions (CH, CD, and CE) and its ethanolic extract (CT) were investigated against Gram-positive bacteria (*Staphylococcus aureus* ATCC 5368), Gram-negative bacteria (*Escherichia coli* ATCC 10536 and *Pseudomonas aeruginosa* ATCC 27853), and fungi (*Candida albicans* ATCC 10231) (Figure 9 and Table 4) with the minimum inhibitory concentration (MIC) determination.

The antimicrobial activity of the extract based on the MIC values was classified as follows: 50–500 µg/mL = strong activity; 600–1500 µg/mL = moderate activity; and >1500 µg/mL = weak activity or inactive [53]. According to the previous classification of *Cliona* sp., the total alcoholic extract (CT), dichloromethane (CD), and ethyl acetate (CE) fractions showed strong antibacterial activity against *S. aureus* only with MIC values of 62.5 ± 0.82, 125 ± 0.58, and 250 ± 0.88 µg/mL, respectively, compared to ciprofloxacin (positive control) with MIC values of 1.56 ± 1.2 µg/mL for Gram-positive bacteria and 3.125 ± 0.89 µg/mL for Gram-negative bacteria. Additionally, CT, CD, and CE are active against *E. coli*, with MIC 125 ± 0.62, 125 ± 0.72, and 250 ± 0.98 µg/mL, respectively. Notably, only CD and CE are the active fractions against *P. aeruginosa* with MIC 125 ± 0.92 and 500 ± 0.32 µg/mL, respectively. Interestingly, the CD fraction is the only fraction that revealed strong antifungal activity against *C. albicans* with MIC 500 ± 0.9 µg/mL compared to the fluconazole (positive control) with MIC values 50 ± 0.24 µg/mL. The significant antimicrobial and cytotoxic activities might be attributed to the presence of different phytosterols (*β*-sitosterol, stigmasterol, coprostanol, and brassicasterol) and sphingomyelin derivatives with reported potent antibacterial activity [54,55].

#### 2.3.2. Antioxidant Activity

**Figure 10:**
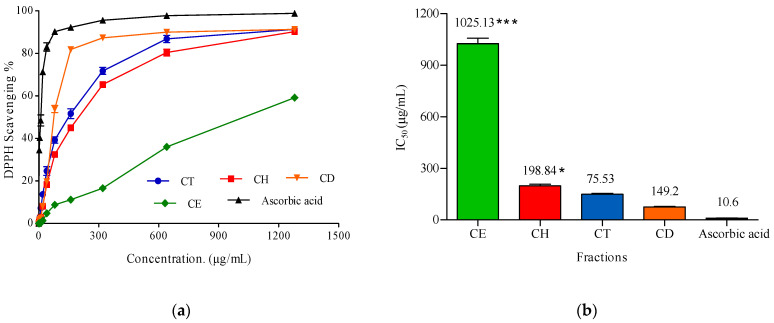
(**a**) 2,2-diphyenyl-picrylhydrazyl (DPPH) radical scavenging activity of different concentrations (5–1280 µg/mL) of *Cliona* sp. Total extract (CT), Hexane (CH), Dichoromethane (CD), and Ethyle acetate (CE) fractions. (**b**) IC_50_ of antioxidant activity of *Cliona* sp. total extract (CT), Hexane (CH), Dichloromethane (CD), and Ethyl acetate (CE) fractions and ascorbic acid (positive control). DPPH in methanol (without the tested sample) was used as a negative control. Data were analysed by using one-way ANOVA and statistical significance was calculated with Dunnett’s multiple comparisons test, and the significance level compared to the control is indicated by asterisks (*, *p* < 0.05; ***, *p* < 0.0001). The data display the mean ±SD of three biological replicas.

Sponges and seaweeds represent an abundant source of natural antioxidants [56]. In the present work, the antioxidant activity of the *Cliona* sp. total extract (CT), *n*-hexane (CH), dichloromethane (CD), and ethyl acetate (CE) fractions are evaluated using the DPPH method and ascorbic acid as a positive control (Figure 10).

The tested extract and its fractions showed a concentration-dependent antioxidant activity by an increase in their DPPH radical scavenging percentage as demonstrated in Figure 10A. The IC_50_ values (the concentration required to scavenge DPPH by 50%) are presented in Figure 10B. Notably, the smaller the IC_50_, the higher the scavenging activity, and it was reported by [46] that the tested extract or fraction is considered a weak antioxidant when the IC_50_ values are in the range of (151–200), moderate when the values are in the range (100–150), strong when the values are in the range (50–100), and very strong when the IC_50_ values are <50. According to the previous classification, the dichloromethane fraction showed strong DPPH scavenging activity with IC_50_ 75.53 ± 3.41 µg/mL, followed by the total extract (CT) and the *n*-hexane fraction (CH) with IC_50_ 149.2 ± 4.85 and 198.84.2 ± 8.23 µg/mL, respectively.

However, the ethyl acetate fraction of the *Cliona* sp. has a very weak antioxidant potential, with IC_50_ 1025.13 ± 32.79 µg/mL, compared to ascorbic acid as a positive control with IC_50_ 10.6 ± 0.8 µg/mL. The activity of the dichloromethane fraction is probably due to the presence of β sitosterol and stigmasterol.

Marine sterols’ antioxidant activity has been demonstrated by their ability to normalize various oxidative markers and to promote the expression of enzymatic and non-enzymatic antioxidants. Furthermore, they have structure and function similarities with biological sterols, especially cholesterol. Stigmasterol and *β*-sitosterol, specifically, have shown very promising results in clinical trials against several diseases [57]. Marine sterols may also provide prospective lead compounds in the development of new therapeutic drugs, due to recent advances in technology such as nanoparticles and microencapsulation [57].

#### 2.3.3. Cytotoxic Activity

**Figure 11:**
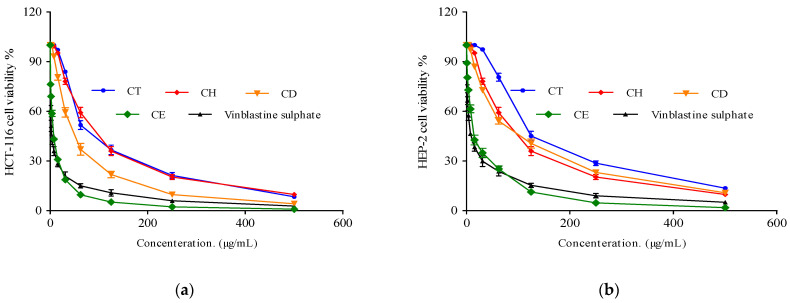
(**a**) Cytotoxic activity of *Cliona* sp. total extract (CT), Hexane (CH), Dichoromethane(CD), and Ethyle acetate (CE) fractions against the HCT-116 cell line at different concentrations. DMSO and vinblastine sulphate were used as negative and positive controls, respectively. (**b**) Cytotoxic activity of *Cliona* sp. total extract (CT), Hexane (CH), Dichoromethane(CD), and Ethyle acetate (CE) fractions against HEP-2 cell line at different concentrations. Data were analyzed by using one-way ANOVA and the statistical significance was calculated with Dunnett’s multiple comparisons test. A *p*-value < 0.05 was considered statistically significant. The data display the mean ± SD of three biological replicates.

In the current study, an MTT assay was used to assess the cytotoxic activity of *Cliona* sp. total extract (CT), *n*-hexane (CH), dichloromethane (CD), and ethyl acetate (CE) fractions against HCT-116 (Colon carcinoma) and HEP-2 (larynx carcinoma) cell lines using a concentration range of 0–500 µg/mL. The potency of the cytotoxic substances based on the IC_50_ values was classified as follows: IC_50_ ≤ 20 µg/mL is highly active, IC_50_ 21–200 µg/mL is moderately active, IC_50_ 201–500 µg/mL is weakly active, and IC_50_ > 501 µg/mL is inactive, which is consistent with the American National Cancer Institute protocol [58]. The tested extract (CT) and fractions (CH, CD, and CE) (Figure 11 and Table 5) showed remarkable cytotoxic activity against the investigated cell lines with an IC_50_ range from 6.11 ± 0.2 to 69.7 ± 3.41 µg/mL for HCT-116 (colon carcinoma) and from 12.6 ± 0.9 to 116.5 ± 4.09 µg/mL for HEP-2 (larynx carcinoma). Interestingly, the *Cliona* sp. ethyl acetate fraction (CE) showed potent cytotoxic activity with IC_50_ 6.11 ± 0.2 and 12.6 ± 0.9 µg/mL for HCT-116 and HEP-2 cell lines, respectively, compared to vinblastine (positive control) with IC_50_ 2.34 ± 0.28 and 6.61 ± 0.59 for HCT-116 and HEP-2, respectively. The total extract, as well as the other fractions, exhibited moderate cytotoxic activity in the following order: CD > CH > CT [58].

According to the UPLC-ESI-MS/MS analysis, the ethyl acetate fraction (CE) revealed the presence of several compounds with promising reported anticancer activity, particularly stachydrine (proline betain) and taurine (2-aminoethanesulfonic acid). Several research studies have demonstrated the potent anticancer properties of stachydrine in a wide range of cancer types, including colon cancer [59], breast cancer [60], gastric cancer [61], and prostate cancer [62], by preventing cell migration and invasion, inhibiting cell proliferation, and triggering apoptosis [63]. In addition, various reports have documented the antioxidant, hypoglycemic, and anti-inflammatory properties of taurine. Taurine not only reduces the chemotherapy’s side effects but also causes cytotoxic activity by preventing cell proliferation and triggering apoptosis [64].

Consistent with our results, several extracts or fractions of sponges collected from the Red Sea have cytotoxic activities such as *Xestospongia testudinaria* [65], *Haliclona* sp. [66], *Spheciospongia vagabunda*, [67], and *Hyrtios erectus* [68].

## 3. Materials and Methods

### 3.1. General Materials and Methods 

A UV lamp was used for thin layer chromatography (TLC) visualization: UVP, GL-58 (λ_max_ 254 and 366 nm). A circulating hot-air oven, WT-binder 7200 (Tuttlingen, Germany), was used in this study.

UPLC-ESI-MS/MS in positive ionization mode was done on a XEVO-TQD triple-quadruple instrument (Waters Corporation, Milford, MA, USA) mass spectrometer; Column, ACQUITY UPLC BEH C_18_ 1.7 mm, 2.1 × 50 mm; column flow rate, 0.2 mL/min; solvent system consisted of (A) water containing 0.1% formic acid, (B) methanol containing 0.1% formic acid (Ain Shams University, Cairo, Egypt).

Nuclear magnetic resonance (NMR) experiments, 1D and 2D analyses, were carried out using a Bruker AMX 400 MHz (Billerica, MA, USA) for ^1^H NMR and with standard pulse sequences operating at 100 MHz for ^13^C-NMR. ^1^H-^13^C one-bond connectivity was detected with the HSQC gradient pulse factor selection. Two- and three-bond connectivities were identified by the HMBC experiment. Coupling constants (*J*) are reported in Hz and chemical shifts are reported in δ (ppm) unless otherwise mentioned. The internal standard is Tetramethylsilane. The spectroscopic grade of DMSO-*d_6_* (solvent at room temperature) was used for spectral analysis.

### 3.2. Collection of Marine Sponge Samples

**Figure 12:**
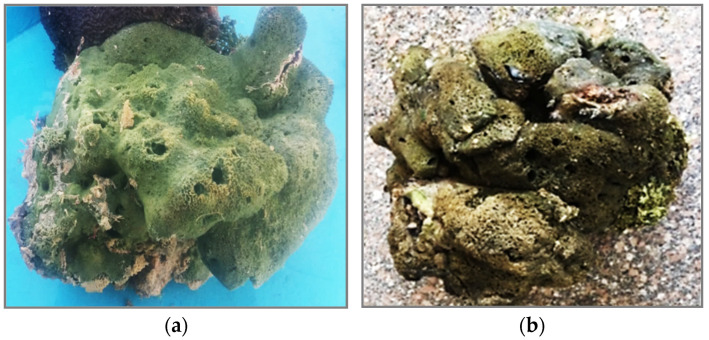
Photographs of *Cliona* sp. Sponge; (**a**) just collected, (**b**) frozen specimen.

Marine sponges, namely *Cliona* sp. Class Demospongiae, were collected from the Red Sea, 20 km away from Sharm El sheikh [27°45′57.8″N 34°22′10.8″E] by scuba diving at a depth of 8:10 m off, during November–December 2018 (Figure 12). The collected material was immediately frozen and kept at −20 °C until investigation. Saad Zakaria, Marine Science Department, Faculty of Science, Suez Canal university defined the sponges’ biomass.

### 3.3. Extraction and Fractionation of Cliona sp.

The fresh sponge material *Cliona* sp. (2840 g wet weight) was frozen immediately after collection. The sponge material was chopped while frozen into small pieces and shed dried for 48 h. It was then extracted with absolute ethanol (3 × 3 L) at room temperature. The combined crude extract was evaporated under vacuum, and the concentrated extract (91.2 g) was dispersed in water/methanol (9:1) and partitioned successively with *n*-hexane, dichloromethane, and ethyl acetate to obtain *n*-hexane, (16.67 g), dichloromethane, (13 g) and ethyl acetate (0.7 g) fractions.

#### 3.3.1. Isolation of Compounds **1** and **2** from n-Hexane Soluble Fraction of *Cliona* sp.

About 15 g of *n*-hexane soluble fraction of *Cliona* sp. was dissolved in the least amount of dichloromethane/methanol and the slurry was transferred to the top of the dry vacuum column (350 g, 8 × 20 cm) packed with silica gel (Merck Silica Gel 60–0.015–0.040 mm). The elution of the column was carried out with *n*-hexane and the polarity was gradually increased using dichloromethane followed by methanol. Fractions eluted with 70% *n*-hexane /CH_2_Cl_2_ were evaporated to afford (3.6 g) residue, which was chromatographed on the silica gel column (50 g, 40 × 2 cm) for purification. The elution was started with *n*-hexane followed by ethyl acetate, and then methanol in a gradient elution manner. Important fractions eluted with 95% *n*-hexane in ethyl acetate were concentrated under a vacuum and subjected to crystallization from hot methanol to afford a mixture of compounds **1** and **2** (93 mg). The mixture was obtained as a white amorphous powder that is freely soluble in dichloromethane.

#### 3.3.2. Isolation of Compound **3** from Dichloromethane Soluble Fraction of *Cliona* sp.

About 11 g of dichloromethane soluble fraction of *Cliona* sp. was placed on the top of the silica gel column. The elution was started with *n*-hexane and the polarity was gradually increased using ethyl acetate followed by methanol. The collected fractions were concentrated under reduced pressure. Compound **3**, (7 mg) crystalline white powder, freely soluble in dichloromethane, was isolated from the fractions eluted with 97% *n*-hexane /CH_2_Cl_2_.

#### 3.3.3. Isolation of Compound **4** from Ethyl Acetate Soluble Fraction of *Cliona* sp.

About 0.5 g of ethyl acetate soluble fraction of *Cliona* sp. was placed on the top of sephadex (LH-20) column and was eluted with *n-*hexane and methanol. Twenty-six fractions (10 mL, each) were collected. Combined fractions (11–12) were concentrated and crystallized using hot methanol/acetone to give 3 mg of colorless needle-shaped crystals soluble in hot methanol designated as compound **4**.

#### 3.3.4. Compounds **1** & **2** (Coprostanol & Brassicasterol)

A total of (93 mg) white amorphous gave one spot with R_f_ 0.54 (solvent system, light petroleum: ethyl acetate 8:2). The ^1^H-NMR (400 MHz, CDCl_3_) ^13^C-NMR-DEPT 135 (100 MHz, CDCl_3_) for compounds **1** (coprostanol) and **2** (Ergosta-5,22-dien-3-ol (brassicasterol) are summarized in Table S1. EI-MS *m*/*z* (relative int. %) 388 (M^+^ 2.98), 373 (1.30), 355 (2.74), 344 (1.59), 331 (1.29), 316 (1.23), and 255 (1.94) for coprostanol (C_27_H_48_O) (Figure S3), in addition to 398 (M^+^ 2.60), 383 (1.65), 355 (2.64), 327 (1.34), 300 (1.56), 299 (1.45), 271 (5.39), and 229 (2.53) for brassicasterol (C_28_H_46_O) (Figure S4).

#### 3.3.5. Compound **3** (Stigmasterol)

A total of 7 mg crystalline white powder was collected with R_f_ 0.36 (solvent system, dichloromethane 100%). ^1^H-NMR (400 MHz, CDCl_3_) and ^13^C-NMR-DEPT 135 (100 MHz, CDCl_3_) are summarized in Table S2. EI-MS (positive ion) of **3** showed *m*/*z*: 413 [M^+^ + H]^+^ and 394 [M^+^ + H-H_2_O]^+^.

#### 3.3.6. Compounds **4** (Taurine)

A total of 3 mg colorless needle crystals were collected with R_f_ 0.59 (solvent system, ethyl acetate: methanol: water, 6:1.5:0.8). Calcd. for C_6_H_6_O_4_. ^1^H-NMR (400 MHz, CDCl_3_) for compounds **4**, δ_H_ 3.26 (H-1), 3.06 (H-2), ^13^C-NMR-DEPT 135 (100 MHz, CDCl_3_) δ_C_ 49.7 (C-1), and δ_C_ 36.6 (C-2).

### 3.4. UPLC-ESI-MS/MS Analysis and Separation Method of Cliona sp. Extract and Its Fractions

*Cliona* sp. extract, and CH, CD, and CE fractions, were analyzed in an ACQUITY UPLC-BEH coupled to a XEVO TQD triple quadruple Mass Spectrometer (Waters Corporation ^®^, Milford, MA, USA), which was equipped with an electrospray ionization (ESI) source functioning with positive polarity at a mass range of *m*/*z* 100–1000 atomic mass units. The tested extract and fractions were prepared at a concentration of 0.2–0.5 mg/mL in HPLC methanol and filtered through a 0.2 µm membrane disc filter, and then 10 µL of the sample was injected. The constituents were separated using a binary LC solvent system controlled by MassLynx software (version 4.1) Waters Corporation (Milford, MA, USA) to analyze the MS and MS^2^ data. The reversed-phase separations were performed by an ACQUITY UPLC-BEH column [silica C_18_, 1.7 µm (particle size), 2.1 × 50 mm (inner diameter), Waters ^®^] eluted with H_2_O + 0.1% formic acid (A) and Methanol+ 0.1% formic acid (B), HPLC grade, at 0.2 mL/min flow rate with the same gradient used by [19,69] as follows: 0–2 min 10% B isocratic; 2–5 min, linear gradient B 10 to 30%; 5–15 min, linear gradient from 30% to 70% B; 15–22 min, linear gradient from 70% to 90% B; 22–25 min, 90% B isocratic. Finally, the process included washing and reconditioning the column. The ESI parameters in positive ionization mode were adjusted as follows: source temperature 150 °C; cone voltage 30 eV; capillary voltage 3 kV; desolvation temperature 440 °C; cone gas flow 50 L/h; and desolvation gas flow 900 L/h. The MS^2^ settings were kept at 30 eV of collision energy.

### 3.5. GC-MS Analysis of Compounds **1** & **2**

Compounds **1** and **2** were subjected to GC/MS analysis according to [70] with minor modification. GC/MS Analysis Mass spectra were recorded using (Shimadzu GCMS-QP2010, Kyoto, Japan) equipped with Rtx-5MS ^®^ fused bonded column (30 m × 0.25 mm i.d.,× 0.25 µm film thickness) (Restek Inc., Edmond, OK, USA) equipped with a split–splitless injector. The initial column temperature was kept at 50 °C for 3 min and programmed to 300 °C at a rate of 5 °C/min and kept constant at 300 °C for 10 min. Injector temperature was 280 °C. Helium carrier gas flow rate was 1.37 mL/min. All the mass spectra were recorded applying the following condition: (equipment current) filament emission current, 60 mA; ion source, 220 °C. Ionization voltage, 70 eV; diluted samples (1% *v*/*v*) were injected with split mode (1:15, split ratio).

### 3.6. Biological Activities

The antioxidant, cytotoxic, and antimicrobial activities of the *Clion sp.* extract and CH, CD, and CE fractions were done at the Regional Center for Mycology and Biotechnology (RCMB) at the Al-Azhar University.

#### 3.6.1. Antioxidant Activity

The antioxidant activity of the CT extract, and CH, CD, and CE fractions, was determined by the DPPH free radical scavenging assay as described by [19,71,72] with minor modifications. Briefly, DPPH (2,2-diphenyl-1-picrylhydrazyl) radical in methanol (0.004% *w*/*v*) was freshly prepared and stored at 10 °C in the dark. Different concentrations (2.5–1280 µg/mL) of the tested extract and its fractions in methanol were also prepared. Forty microliters of aliquot of the methanol solution was added to 3 mL of DPPH solution and allowed to stand for 10 min at room temperature in the dark. Absorbance measurements were recorded immediately with a UV-visible spectrophotometer (Milton Roy, Spectronic 1201, Houston, TX, USA). The decrease in absorbance at λ_max_ 515 nm was measured continuously, with data being recorded at 1 min intervals until the absorbance stabilized (16 min). The absorbance of the DPPH radical without antioxidant (negative control) and the reference compound ascorbic acid (positive control) were also measured. All the determinations were performed in three replicates and averaged. The percentage inhibition (PI) of the DPPH radical was calculated according to the equation:

PI = [(*A*C − *A*T)/AC] × 100 (1)

where *A*C = absorbance of the control at t = 0 min and *A*T = absorbance of the sample + DPPH at t = 16 min [73]. The DPPH radical scavenging percentage was plotted against each sample concentration and ascorbic acid (µg/mL) to measure the antioxidant capacity (IC_50_), which resulted in a 50% reduction in the DPPH solution absorbance from its initial absorbance. The lower the IC_50_, the stronger antioxidant activity.

#### 3.6.2. Cytotoxic Activity

*Cliona* sp. extract, and CH, CD, and CE fractions, were evaluated for their cytotoxic activity in two cell lines against HEB-2 and HCT-116 (Human larynx carcinoma and Colon carcinoma) using MTT assay as explained by [19,74,75]. Vinblastine sulphate and DMSO (Dimethyl sulfoxide) were used as positive and negative controls, respectively. The tested cell lines were obtained from VACSERA Tissue Culture Unit Giza, Egypt. DMEM (Dulbecco’s Modified Eagle’s Medium) was used for the tested cells’ propagation. Stock solutions of the extract and fractions were prepared in 10% DMSO in ddH_2_O. The cytotoxicity was determined using the MTT assay as described by [19,75].

In brief, cells were seeded in 96-well plates (100 µL/well at a density of 1 × 10^4^ cells/mL) and incubated in 5% CO_2_ at 37 °C for 24 h. Cells were treated in triplicate with various concentrations of the tested extract and fractions after 24 h. The viable cell yield was determined by a colorimetric method as follows: after additional 24 h, the supernatant was removed and a crystal violet solution (1%) was added to each well for at least 30 min. Then, the stain was removed and the plates were washed using tap water until all the excess stains were removed. Next, 30% Glacial acetic acid was added to the whole wells and mixed. The absorbance of the plates was measured after gently shaking the Microplate reader (TECAN, Inc., Morrisville, NC, USA), using a test wavelength of 490 nm. Compared with the untreated cells, the optical density was measured with the microplate reader (SunRise, TECAN, Inc, Morrisville, NC, USA) to determine the viable cell numbers. The following equation was used to determine the percentage of viability.

Cell viability % = [1 − (ODt/ODc)] × 100%(2)

where ODt is the mean optical density of wells treated with the tested sample and ODc is the mean optical density of the untreated cells. The relation between the surviving cells and drug concentration was plotted to obtain the survival curve of each tumor cell line after treatment with the specified extract or fraction. The concentration required to cause toxic effects in 50% of the intact cells (IC_50_) was estimated from the graphic plots of the dose response curve for each concentration using the Graphpad Prism 5 software (Graphpad software, San Diego, CA, USA). 

#### 3.6.3. Antimicrobial Activity

The *Cliona* sp. extract and its subfractions’ (CH, CD, and CE) antibacterial activities were investigated by using the well diffusion method, as described by [19,76], against *Staphylococcus aureus* (*S. aureus*, ATCC 5368) as Gram-positive bacteria and *Escherichia coli* (*E. coli*, ATCC 10536) and *Pseudomonas aeruginosa (P. aeruginosa*, ATCC 27853) as Gram-negative bacteria. The activity was determined by measuring the inhibition zone diameter in mm from 3 independent experiments and the average was considered. Ciprofloxacin (100 µg/mL) was used as a standard antibacterial drug (Positive control). The antifungal activity was also studied against *Candida albicans* (*C. albicans,* ATCC 10231) as reported by [19]; Fluconazole (100 µg/mL) was used as a positive antifungal control. The tested samples were dissolved in dimethyl sulfoxide (DMSO) at a concentration of 500 µg/mL. DMSO was used as a negative control. The Muller Hinton Agar (MHA) medium was used for bacterial strains and the PDA (Potato Dextrose Agar) medium was used for *c. albicans*. The wells were filled with 100 µL of the stock solution of each sample, and the standards and DMSO Cultures were incubated at 37 °C for 24–48 h for fungi and 14–18 h for bacteria [77,78]. The tested bacterial strains and *candida albicans* were obtained from the Regional Center for Mycology and Biotechnology (RCMB) at Al-Azhar University, Egypt.

##### Minimum Inhibitory Concentration Determination (MIC)

The *Cliona* sp. extract and its subfractions’ (CH, CD, and CE MIC) values were determined using the agar dilution method as explained by [19]. In summary, the CT extract and its CH, CD, and CE fractions were dissolved in DMSO (not more than 5% and 2.5% for bacteria and fungi, respectively) and serially diluted. A series of MHA plates (for the tested bacterial strains) and PDA plates (for *C. albicans*) containing different dilutions of each extract and fraction were prepared. The tested bacterial strains were grown overnight on MHA, and purified colonies were suspended in 0.9% saline. The bacterial inoculum turbidity was adjusted to 0.5 McFarland standard (2.5 × 10^8^ cfu /mL) and then diluted to 1:10 with sterile saline. The prepared MHA plates were inoculated by delivering 2 µL of the prepared inoculum on their surfaces to obtain a final concentration of 10^4^ cfu per spot [79]. *C. albicans* was streaked on PDA, and the purified colonies were suspended in saline. The turbidity was adjusted to 0.5 Mcfarland standard (5 × 10^6^ cfu/mL) and was then diluted in a 1:10 dilution with saline. Prepared PDA containing different concentrations of CT extract and CH, CD, and CE fractions were inoculated by delivering 2 µL of the prepared inoculum; therefore, the final concentration of the inoculum was 10^3^ per the produced spot. Inoculated plates were incubated at 30 °C for 24–48 h and were examined for the presence of microbial growth. MIC is the lowest concentration of the antimicrobial agent that inhibits growth completely [80,81].

### 3.7. Statistical Analysis

GraphPad Prism 5 software (GraphPad Software, San Diego, CA, USA) was used for plotting the collected data. One-way ANOVA followed by Dunnett’s multiple comparisons test was used for the data analysis and statistical significance calculation. A *p*-value < 0.05 was considered statistically significant. The data show the mean ± SD of three biological replicas.

## 4. Conclusions

In the present study, the *Cliona* sp. total extract, and the dichloromethane and ethyl acetate fractions were investigated using the UPLC-ESI-MS/MS analysis, which revealed the tentative identification of **23**, **16**, and **24** compounds from the *n*-hexane (CH), dichloromethane (CD), and ethyl acetate (CE) fractions of *Cliona* sp., respectively. Isolation and structure confirmation revealed four major compounds (coprostanol, brassicasterol, stigmasterol, and taurine) from the previous fractions. The cytotoxic, antioxidant, and antimicrobial activities of the ethanolic total extract and its subfractions were also determined in vitro. Remarkably, ethyl acetate was the most potent cytotoxic fraction, while dichloromethane showed a broad antimicrobial activity against all of the tested strains. Notably, the *Cliona* sp. total ethanolic extract is very active against the Gram-positive bacteria *S. aureus*.

In conclusion, this is the first study that investigates the chemical composition and biological activities of *Cliona* sp. from the Red Sea, Egypt. Based on the previous results, the *Cliona* sp. extract and its fractions could be a promising source of potent cytotoxic, antioxidant, and antimicrobial natural agents for multidrug-resistant bacterial and fungal strains, as well as different cancers. Further studies are planned for the most potent fractions of this sponge to test their effects against different cancer cell lines and resistant microbial strains. Further studies are also planned to isolate and identify the bioactive compounds from each major fraction using advanced techniques such as preparative or semipreparative HPLC.

## Figures and Tables

**Table 1:** Metabolites tentatively identified from *n*-hexane fraction using UPLC-ESI-MS/MS analysis in positive ionization mode.

No.	Compound Name	R_t_ (min.)	Parent Ion (*m*/*z*)	MS^2^ Fragments (*m*/*z*)	Area % Total	Reference
1	Asparagine	0.48	133 [M + H]^+^	88, 73, 59, 44	2.40	[26]
2	Asparagine isomer	0.66	133 [M + H]^+^	88, 73, 59, 44	1.30	[26]
3	Asparagine isomer	0.67	133 [M + H]^+^	88, 73, 59, 44	1.20	[26]
4	l-Serine	0.72	104 [M + H]^+^	60, 59, 44	3.66	[26]
5	l-Serine isomer	0.83	104 [M + H]^+^	60, 59, 44	1.09	[26]
6	Dibutyl Phthalate	18.18	301 [M + Na]^+^	149	1.89	[27,28]
			279 [M + H]^+^			
7	Sphingomyelin derivative	27.38	482 [M + H]^+^	184, 124, 104, 60	7.62	[29]
8	Aflatoxin G2	27.74	353 [M + H]^+^	295, 275, 257, 244	7.62	[30,31,32]
9	Sphingomyelin derivative	27.79	508 [M + H]^+^	184, 124, 104, 60	6.07	[29]
10	Cyclopeptine	28.08	282 [M + 2H]^+^	134, 120, 224, 57	1.89	[33]
11	Sphingomyelin derivative	29.07	511 [M + H]^+^	184, 124, 104, 60	22.6	[29]
12	Dioctyl Phthalate	29.20	413 [M + Na]^+^	279, 149	10.65	[27,28]
			391 [M + H]^+^			
13	Sphingomyelin derivative	29.52	511 [M + H]^+^	184, 124, 104, 60	22.6	[29]
14	Sphingomyelin derivative	30.65	525 [M + H]^+^	184, 124, 104, 60	8.47	[29]
15	Sphingomyelin derivative	31.18	525 [M + H]^+^	184, 124, 104, 60	1.29	[29]
16	(2*S*,3*R*,4*E*)-2-(14´-methyl-pentadec anoylamino)-4-octadecene-l,3-diol	31.19	539 [M + H]^+^	353, 282, 264, 126	0.74	[34]
17	Ceramide sphingoid base d18:1	33.21	476 [M + H]^+^	329, 299, 281, 215, 126	0.50	[34]
	Fatty acid base C12:3					
18	*N*-heptadecanoyl sphingosine	34.09	551 [M + 2H]^+^	299	2.69	[35]
19	Undecylenic phytosphingosine C11:1	35.73	483 [M + H]^+^	317, 255	1.94	[35]
20	α-Tocopherol	36.77	431 [M + H]^+^	377, 190, 177, 149, 136, 120	2.04	[36,37]
21	Docosanoyl sphingosine	36.78	648 [M + H]^+^	365, 125, 97, 71	0.86	[34,38]
	Sphingoid base d22:0					
	Fatty acid base C20:1					
22	Coprostanol *	37.0	371 [M + H-H_2_O]^+^		22.06	[39],
23	Brassicasterol *	37.43	399 [M + H]^+^	381	1.00	[21]

* Compounds isolated from *n*-hexane fraction.

**Table 3:** Metabolites tentatively identified from ethyl acetate fraction using UPLC-ESI-MS/MS analysis in positive ionization mode.

No.	Compound Name	R_t_ (min.)	Parent Ion (*m*/*z*)	MS^2^ Fragments (*m*/*z*)	Area % Total	Reference
1	Glutamine	0.25	142 [M + H]^+^	101, 56	5.94	[26]
2	Taurine *	0.37	127 [M + 2H]	97, 43	4.90	[46]
3	Stachydrine	0.76	144 [M + H]^+^	153, 99	16.84	[47]
4	Maleimide 5-oxime	0.86	127 [M + H]^+^	110, 84, 82, 44	2.31	[18]
5	1-Pyrroline-5-carboxylic acid	1.10	114 [M + H]^+^	69, 42	0.69	[26]
6	Valine	1.24	118 [M + H]^+^	100, 72, 56	0.28	[48]
7	2-Aminocylohexane carboxylic acid	1.27	144 [M + H]^+^	128, 98	1.47	[49]
8	Tetillapyrone	1.39	243 [M + H]^+^	197, 123, 86	0.83	[18]
9	Cysteine	1.43	122 [M + H]^+^	104	3.92	[50]
10	Stachydrine isomer	3.02	144 [M + H]^+^	153, 99	0.37	[47]
11	Tetillapyrone isomer	3.24	485 [2M + H]^+^	243 [M + H]^+^, 197, 131	2.64	[18]
12	Hydroxy proline	4.28	132 [M + H]^+^	86, 56	0.83	[51]
13	5-Sulfo-l-cysteine	5.02	202 [M + H]^+^	156	0.28	[26]
14	Allo-isoleucine	5.04	132 [M + H]^+^	86, 69 57.18, 41	2.94	[26]
15	Isoleucine derivative	5.05	216 [M + H]^+^	132, 86.	4.22	[26]
16	Isoleucine	5.35	132 [M + H]^+^	86, 69, 41	2.94	[26]
17	l-3-(3,4 Dihydroxyphenyl)-alanine (Dopa)	6.40	198 [M + H]^+^	139, 111	0.43	[26]
18	Glycerol ether	6.40	313 [M - H]^−^	227, 116	2.20	[18]
19	*N*-methyl tryptophan derivative	7.19	309 [M]^+^	219	2.64	[52]
20	Glycerol ether	7.33	345 [M + H]^+^	252, 201, 158 [M + H-(CH_2_)2-OCH_3_]	0.58	[18]
21	*N*-methyl tryptophan	8.92	219 [M + H]^+^	188, 173, 118, 101	1.40	[52]
22	*N*-methyl tryptophan isomer	9.44	219 [M + H]^+^	188, 173, 118, 101	0.50	[52]
23	Orotidine	11.11	289 [M + H]^+^	270, 252, 132, 89	0.63	[26]
24	Tetradehydro tetillapyrone	12.03	239 [M + H]^+^	193, 113, 85	8.15	[18]

* Compounds isolated from ethyl acetate fraction.

**Table 4:** Antimicrobial activity of *Cliona* sp. Total extract (CT), Hexane (CH), Dichloromethane (CD), and Ethyl acetate (CE) fractions by agar diffusion method.

Microorganism/Extract		Inhibition Zone (IZ mm) Diameter (Mean ± SD)/Minimum Inhibitory Concentration (MIC µg/mL)							
		Gram-Positive Bacteria		Gram-Negative Bacteria				Fungi	
		*Staphylococcus Aureus* ATCC 5368		*Escherichia Coli* ATCC 10536		*Pseudomonas aeruginosa* ATCC 27853		*Candida Albicans* ATCC 10231	
		IZ	MIC	IZ	MIC	IZ	MIC	IZ	MIC
*Cliona* sp.	Total extract (CT)	21 ± 0.72	62.5 ± 0.82	22 ± 0.72	125 ± 0.62	-	-	23 ± 0.92	>3000 ± 1.7
	Hexane (CH)	18 ± 0.82	2000 ± 1.4	21 ± 0.59	>2000 ± 1.3	-	-	18 ± 0.63	>3000 ± 1.2
	Dichloromethane (CD)	26 ± 0.72	125 ± 0.58	25 ± 0.42	125 ± 0.72	25 ±0.42	250 ± 0.92	28 ± 0.52	500 ± 0.9
	Ethyl acetate (CE)	22 ± 0.69	250 ± 0.88-	23 ± 0.83	250 ± 0.98	22 ±0.62	500 ± 0.32	25 ± 0.59	3000 ± 0.38
	Ciprofloxacin	-	1.56 ± 1.2	-	3.125 ± 0.89	-	3.125 ± 0.24	-	-
	Fluconazole	-	-	-	-	-	-	42 ± 0.58	50 ± 0.24
	DMSO (Solvent)	10	-	10	-	19	-	12	-

MIC: 50–500 μg/mL (strong activity), 600–1500 μg/mL (moderate activity), >1500 μg/mL (weak activity) [53,55]. IZ—Inhibitin Zone, MIC—Minimum Inhibitory Concenteration.

**Table 5:** Half maximum inhibitory concentration (IC_50_) of *Cliona* Sp. Total extract (CT), Hexane (CH), Dichloromethane (CD), and Ethyl acetate (CE) fractions in cell viability of HCT-116 and HEP-2 cells after the treatment for 48 h, as measured by MTT assay. The data are presented as µg/mL.

Cell Line	IC_50_ (µg/mL)				
	Tested Total Extract of *Cliona* sp. and Its Fractions				
	CT	CH	CD	CE	Vinblastine Sulphate
HCT-116 (Colon carcinoma)	69.7 ± 3.41	58.4 ± 2.19	44.4 ± 2.6	6.11 ± 0.2	2.34 ± 0.28
HEB-2 (Human Larynx carcinoma)	116.5 ± 4.09	87.5 ± 3.75	82.1± 5.2	12.6 ± 0.9	6.61 ± 0.59

## Data Availability

Not applicable.

## Supplementary Materials

The following supporting information can be downloaded at: https://www.mdpi.com/article/10.3390/molecules28041643/s1, Table S1: ^1^H-NMR (400 MHz, CDCl_3_) ^13^C-NMR-DEPT 135 (100 MHz, CDCl_3_) for compounds **1** & **2**. Table S2: ^1^H-NMR (400 MHz, CDCl_3_) ^13^C-NMR-DEPT 135 (100 MHz, CDCl_3_) HMBC spectral data for compound **3**. Figure S1: EI-MS spectrum of compounds **1** & **2**, Figure S2: GC-MS chromatogram of compounds **1** & **2**, Figure S3: GC-MS spectrum of compound **1**, Figure S4: GC-MS spectrum of compound **2**, Figure S5: ESI-MS/MS spectrum of compound **3**, Figure S6: ESI-MS/MS Spectrum of compound **4**.
