# Supplementary material for: Metabolites Profiling and In Vitro Biological Characterization of Different Fractions of Cliona sp. Marine Sponge from the Red Sea Egypt
Source: Molecules. 2023 Feb 8;28(4):1643. doi: 10.3390/molecules28041643 (PMC9966995; doi:10.3390/molecules28041643)
Supplement: Supplementary file 1 [file molecules-28-01643-s001.zip › molecules-2192619-supplementary.pdf]

Supplementary Materials

Metabolites Profiling and In Vitro Biological Characterization of Different Fractions of Cliona sp. Marine Sponge from the Red Sea Egypt

Wafaa H. B. Hassan [1], Zeinab I. El Sayed [1, 2], Lamya H. Al-Wahaibi [3], Mahmoud M. Abdel-Aal[1], Wael M. Abdel-Mageed [4, 5,*], Eman Abdelsalam [1], and Sahar Abdelaziz [1,*]

[1] Department of Pharmacognosy, Faculty of Pharmacy, Zagazig University, 44519 Zagazig, Egypt

[2] Department of Pharmacognosy, Faculty of Pharmacy, Sinai University, Al-Arish Branch, North Sinai, Egypt

[3] Department of Chemistry, College of Science, Princess Nourah Bint Abdulrahman University, P.O. Box 84428, Riyadh 11671, Saudi Arabia

[4] Department of Pharmacognosy, College of Pharmacy, King Saud University, P.O. Box 2457, Riyadh 11451, Saudi Arabia

[5] Department of Pharmacognosy, Faculty of Pharmacy, Assiut University, Assiut 71526, Egypt.

* Correspondence: wabdelmageed@ksu.edu.sa (W.M.A.); sah_abdelaziz@zu.edu.eg (S.A.)

List of contents

Figure S1: EI-MS spectrum of compounds 1 & 2 (Coprostanol & Brassicasterol).

Figure S2: GC-MS chromatogram of compounds 1 & 2 (Coprostanol & Brassicasterol)

Figure S3: GC-MS spectrum of Coprostanol.

Figure S4: GC-MS spectrum of Brassicasterol.

Figure S5: ESI-MS/MS spectrum of compound 3 (Stigmasterol)

Figure S6: ESI-MS/MS Spectrum of compound 4 (Taurine).

Table S1: ^{1}H-NMR (400 MHz, $CDCl_3$) ^{13}C-NMR-DEPT 135 (100 MHz, $CDCl_3$) for compounds 1 (Coprostanol) and 2 (Ergosta-5,22-dien-3-ol (Brassicasterol)).

Table S2: ^{1}H-NMR (400 MHz, $CDCl_3$) ^{13}C-NMR-DEPT 135 (100 MHz, $CDCl_3$) HMBC spectral data for compound 3 (Stigmasterol).

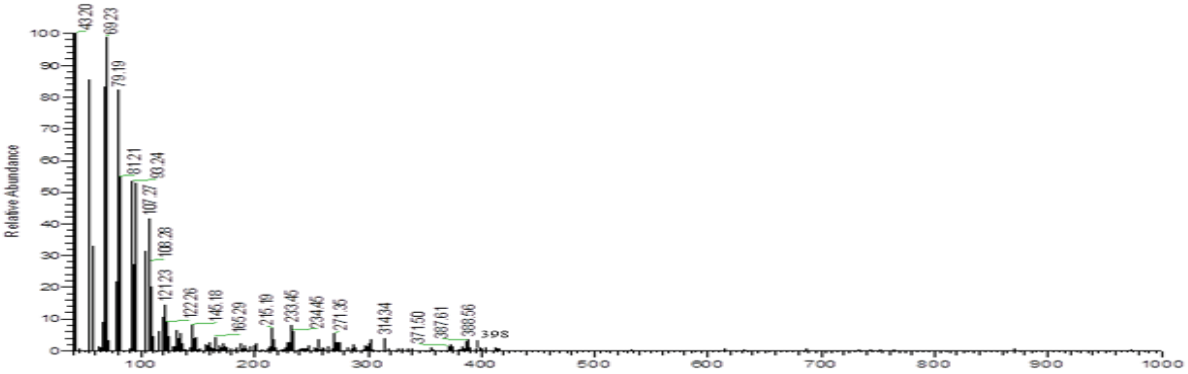

Figure S1: EI-MS spectrum of compounds 1 & 2 (Coprostanol & Brassicasterol)

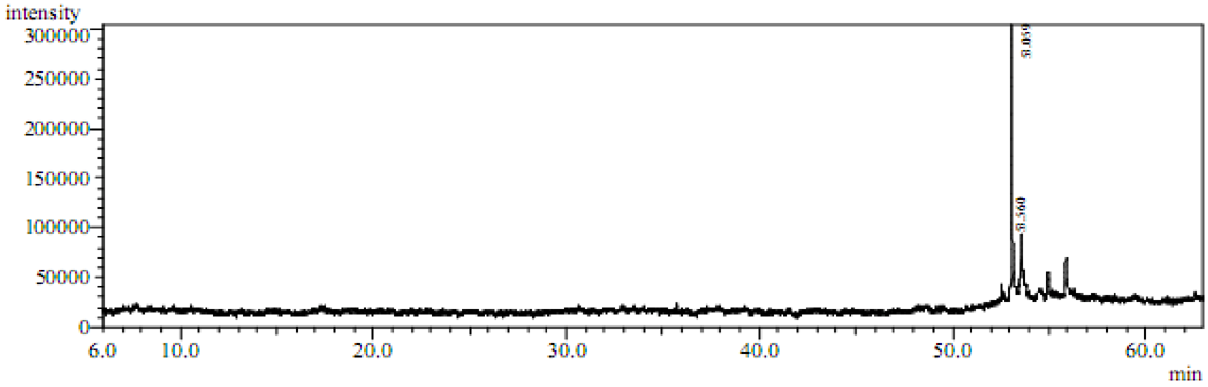

Figure S2: GC-MS chromatogram of compounds 1 & 2 (Coprostanol 88.5% & Brassicasterol 11.5%)

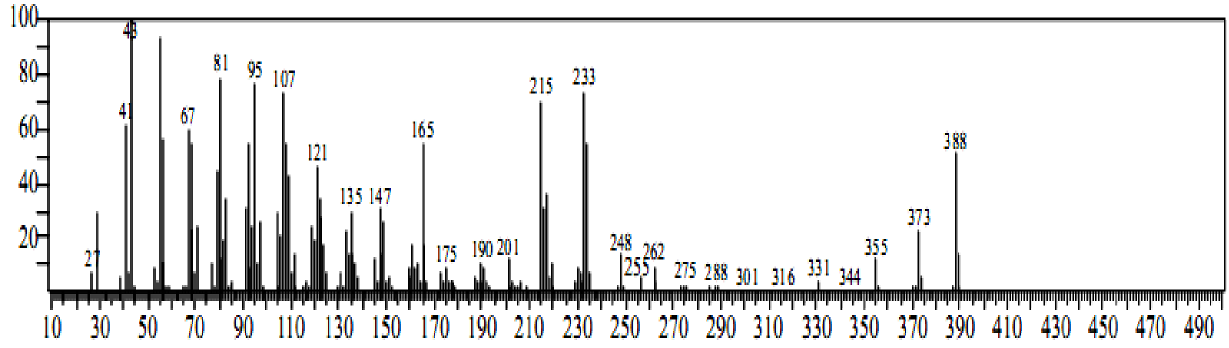

Figure S3: GC-MS spectrum of Coprostanol

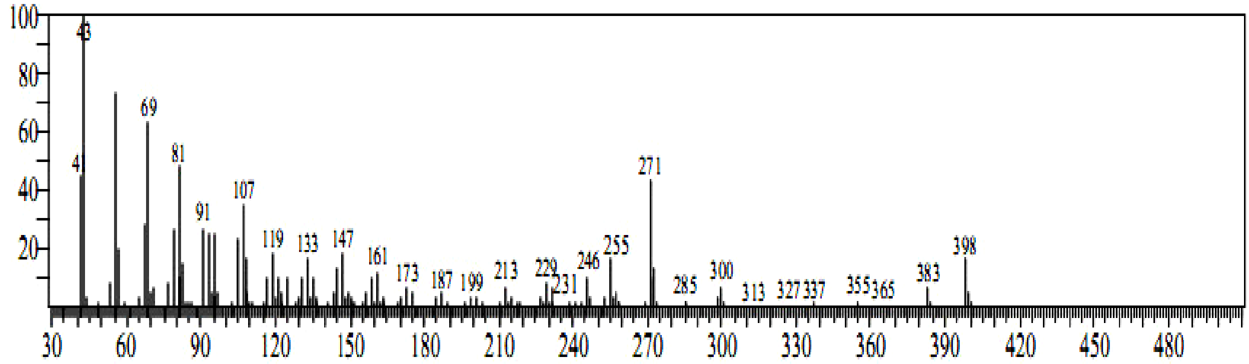

Figure S4: GC-MS spectrum of Brassicasterol

Table S1. ^{1}H-NMR (400 MHz, $CDCl_3$) ^{13}C-NMR-DEPT 135 (100 MHz, $CDCl_3$) for compounds 1 (Coprostanol) and 2 (Ergosta-5,22-dien-3-ol (Brassicasterol)) in comparison with reference

No.	Compound 1 (coprostanol)		Compound 2 Ergosta-5,22-dien-3-ol (brassicasterol)	
	δ_H, ppm [mult., *J*(Hz)]	δ_c, ppm (mult.)	δ_H, ppm [mult., *J*(Hz)]	δ_c, ppm (mult.)
**1**	1.58	35.5, t	1.32	31.3, t
**2**	1.72	31.5, t	1.51	37.2, t
**3**	3.55, (m)	71.4, d	3.61, (m, 1H)	71.8, d
**4**	1.66	37.0, t	2.20	42.3, t
**5**	1.13	39.9, d	--	**141.5, s**
**6**	1.64	28.8, t	5.17 (t, *J* = 8 Hz)	121.7, d
**7**	1.66	31.9, t	2.20	31.9, t
**8**	1.13	35.5, d	1.27	32.1, d
**9**	1.00	54.8, d	1.17	50.2, d
**10**	--	35.5, s	--	37.3, s
**11**	1.65	21.0, t	1.64	21.5, t
**12**	1.56	40.3, t	1.56	40.2, t
**13**	--	42.3, s	--	43.1, s
**14**	1.00	56.5, d	1.03	56.9, d
**15**	1.94	24.3, t	1.94	28.8, t
**16**	1.97	28.3, t	1.94	25.4, t
**17**	1.10	56.3, d	1.23	56.5, d
**18**	0.66, (s)	12.5, q	0.85 (s)	12.3, q
**19**	0.94, (s)	12.4, q	1.01(s)	20.0, q
**20**	1.32	35.8, d	1.99	40.2, d
**21**	0.89, (s)	19.4, q	0.88 (s)	20.3, q
**22**	1.21	36.2, t	5.35 [d, J = 4Hz]	131.8, d
**23**	1.26	25.4, t	5.36 [d, *J* = 4Hz]	136.1, d
**24**	1.21	28.0, t	1.99	43.07, d
**25**	1.64	40.2, d	1.63	33.1, q
**26**	0.92, (s)	23.0, q	0.92 (s)	20.8, q
**27**	0.92, (s)	23.0, q	0.92 (s)	19.5, q
**28**			0.89 (s)	20.8, q

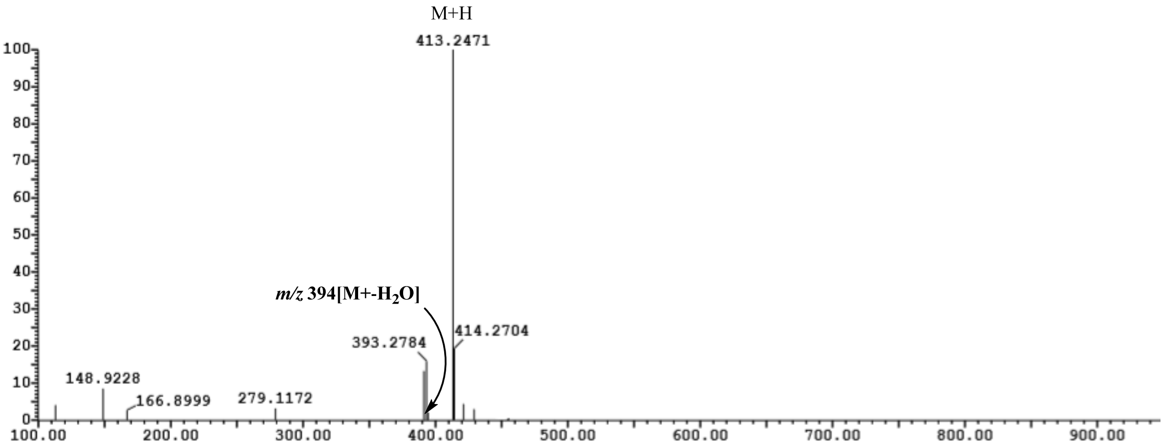

Figure S5: ESI-MS/MS spectrum of compound 3 (Stigmasterol)

Table S2: ^{1}H-NMR (400 MHz, $CDCl_3$) ^{13}C-NMR (100 MHz, $CDCl_3$) for compound 3 (Stigmasterol)

No.	δc , ppm (mult.)	δH, ppm [mult., *J*(Hz)]	No.	δc , ppm (mult.)	δH, ppm [mult., *J*(Hz)]
**1**	37.4 (t)	1.32	**16**	28.83 (t)	1.95
**2**	31.75 (t)	1.51	**17**	56.45 (d)	1.11
**3**	71.79 (d)	3.53	**18**	12.51 (q)	1.29
**4**	42.69 (t)	2.33	**19**	19.23 (q)	1.14
**5**	141.1 (s)	---	**20**	40.13 (d)	1.12
**6**	121.28 (d)	5.53 (t, *J* = 4 Hz)	**21**	20.42 (q)	1.10
**7**	32.18 (t)	2.15	**22**	137.5 (d)	5.40 (d, *J* = 4 Hz)
**8**	31.99 (d)	1.27	**23**	129.28 (d)	5.40 (d, *J* = 4 Hz)
**9**	50.22 (d)	1.23	**24**	50.22 (d)	1.55
**10**	37.09 (s)	---	**25**	35.5 (d)	0.99
**11**	22.9 (t)	1.63	**26**	19.4 (q)	0.84
**12**	40.13 (t)	1.55	**27**	21.1 (q)	0.88
**13**	42.93 (s)	---	**28**	24.31 (t)	1.08
**14**	56.59 (d)	---	**29**	12.2 (q)	0.85
**15**	24.31 (t)	1.95			

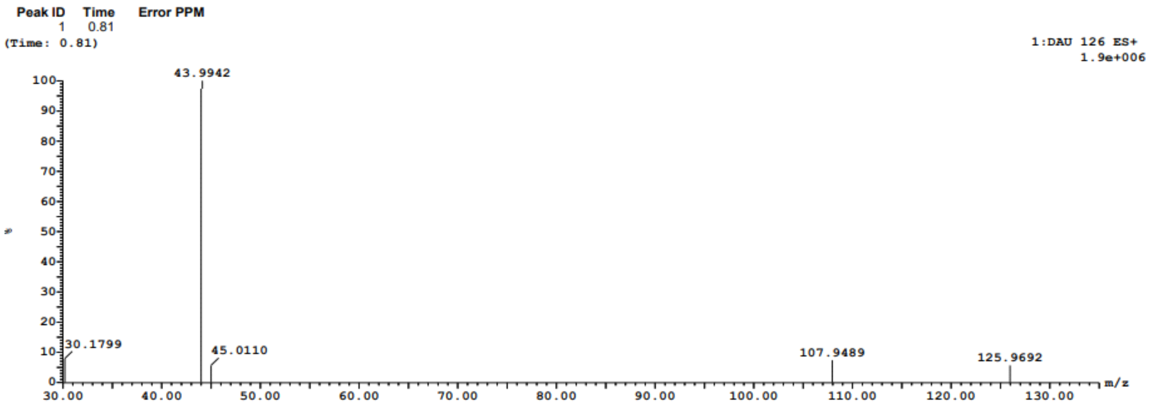

Figure S6: ESI-MS/MS Spectrum of compound 4 (Taurine)
